# The tridecaptins: non-ribosomal peptides that selectively target Gram-negative bacteria†

**DOI:** 10.1039/d0md00413h

**Published:** 2021-01-22

**Authors:** Samantha J. Bann, Ross D. Ballantine, Stephen A. Cochrane

**Affiliations:** School of Chemistry and Chemical Engineering, Queens University Belfast David Keir Building, Stranmillis Road Belfast BT9 5AG UK s.cochrane@qub.ac.uk

## Abstract

Tridecaptins are a re-emerging class of non-ribosomal antibacterial peptides (NRAPs) with potent activity against highly problematic strains of Gram-negative bacteria. An intricate mode of action has been reported to explain the bactericidal activity of these NRAPs, wherein they bind selectivity to the Gram-negative version of the peptidoglycan precursor lipid II on the outer leaflet of the inner membrane and disrupt the proton-motive force. Tridecaptins are highly amenable to synthetic modification owing to their linear structure, therefore, various synthetic analogues have been reported, several of which have enhanced antimicrobial activity, reduced cost of synthesis and/or improved stability towards d-peptidase mediated hydrolysis. It has also been demonstrated that unacylated tridecaptins can act synergistically with clinically relevant antibiotics by sensitizing the outer membrane. This review will summarize past literature on the development/discovery of novel tridecaptin analogues (up until the end of 2020), some of which may be useful therapeutic agents to treat insidious Gram-negative bacterial infections.

## Abstract 2

Tridecaptins are a re-emerging class of non-ribosomal antibacterial peptides (NRAPs) with potent activity against highly problematic strains of Gram-negative bacteria.

## Graphical abstract

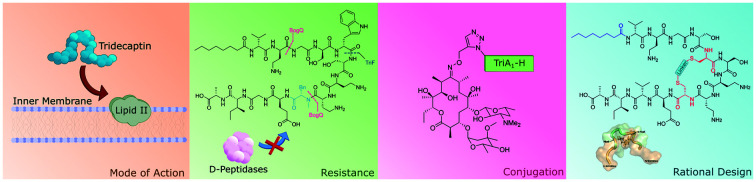

## 1. Introduction

Antimicrobial resistance (AMR) poses a major threat to healthcare systems and global economies and if overlooked, could have disastrous implications for modern medicine. Current projections paint an unsavoury picture; it is predicted that by 2050, 10 million people could die annually due to AMR.^1^ Tuberculosis (TB) claims 1.8 million lives each year^2^ and despite available therapies, approximately half a million cases of TB were reported in 2018 in which the infection was resistant to the front-line antibiotic, rifampicin.^3^ Although the imminent threat of AMR is clear, persistent overuse of antibiotics^4,5^ and a discovery void, which has seen many pharmaceutical companies withdraw from antimicrobial research and development,^6,7^ have perpetuated this issue.

The need for novel antimicrobial agents is clear. While there are many clinically approved antibiotics with selectivity for Gram-positive bacteria,^8^ there remains an urgent demand for those with broader activity against the more robust Gram-negative bacteria. The latter are inherently more difficult to kill than Gram-positives, owing to the presence of an outer membrane (OM) in their structure. This extra layer of protection obstructs membrane crossing for larger antibiotics,^9^ thereby making discovery of Gram-negative-targeting agents more challenging. The ever-increasing prevalence of multidrug-resistant (MDR) bacteria is recognized by the World Health Organisation (WHO) as an imminent threat to human life, particularly the critical priority pathogens *Acinetobacter baumannii*, *Pseudomonas aeruginosa* and *Enterobacteriaceae*,^10^ all of which are Gram-negative pathogens. Fortunately, the current preclinical pipeline of antibacterial research is working towards addressing this issue, as a significant proportion of current research is towards treatments for Gram-negative bacterial infections.^7^

Peptides are experiencing ongoing interest as potential antibacterial candidates; as of 2019, 27 antimicrobial peptides (AMPs) were undergoing clinical trials, while 34 were under preclinical investigation.^11^ AMPs also offer the unique advantage that their often non-specific mechanism of action means resistance is less likely to arise^11–13^ compared to other classes of antibacterial agents, while their relative ease of synthesis makes them favourable antibacterial drug candidates. Non-ribosomal antibacterial peptides (NRAPs) are a promising class of antibiotic drug candidate owing to the nature of their synthesis. Produced by non-ribosomal peptide synthetases (NRPSs), the ability to incorporate both proteinogenic and non-proteinogenic amino acids into the peptide sequence, and the occurrence of enzyme-mediated modifications, allows for diverse structural scaffolds^14,15^ that can facilitate chemical modification to improve efficacy.

The tridecaptins are a linear class of NRAP that display selective activity against Gram-negative bacteria. They were initially discovered in 1978^16^ but after this early work by Kato *et al.*, there was a significant innovation gap until 2014, when research into this class of NRAPs by the Vederas group began.^17^ This review will summarize research relating to the tridecaptins up until November 2020. We have provided the structures of all known natural and synthetic tridecaptin derivatives, as well as any available antimicrobial activity and toxicity data in the supporting information. We recommend that readers refer to this document whilst reading our review.

In early work by Kato *et al.*, three separate antimicrobial tridecapeptides were identified as tridecaptin A, B and C, with differences in their amino acid compositions and lipid tail structures postulated (Table 1). Edman degradation and acid hydrolysis were used to distinguish the structural components of each novel peptide. Tridecaptin A_1_ (TriA_1_) was reported to bear a 3-hydroxy-6-methyloctanoyl (*ai*C_9_h^3^) tail,^20^ while subsequent HPLC analysis identified a major and minor component, differing in amino acid composition. The major component of the native peptide, A_1_ (formerly A_α_) (**1**), contains *allo*-Ile at position 12, while in the minor component, A_2_ (formerly A_β_) (**2**), this residue is replaced with Val12.^18^ Tridecaptins B and C were also reported to possess differing elemental compositions; both have l-Ser13 in place of l-Ala and the former contains a 6-methyloctanoyl (*ai*C_9_) tail while the latter was reported to contain two fatty acyl derivatives, methyl β-hydroxy *anteiso* undecanoate (*ai*C_11_h^3^) and methyl β-hydroxy isodecanoate (iC_10_h^3^).^21^ HPLC analysis also identified differing moieties within TriB and C. Tridecaptin B is comprised of B_α_ (**5**), B_β_ (**6**), B_γ_ (**7**), and B_δ_ (**8**) components, differing only in the presence of Val, Ile or *a*Ile residues (Table 1) whilst tridecaptin C is made up of C_α1_ (**9**), C_α2_ (**10**), and C_β1_ (**11**) distinguishable in both their lipid tails and Val/*a*Ile ratios.^18^

**Table 1:** Original tridecaptin isolates from *Bacillus polymyxa* and additional TriA_3_ and A_4_ variants reported by the Vederas group

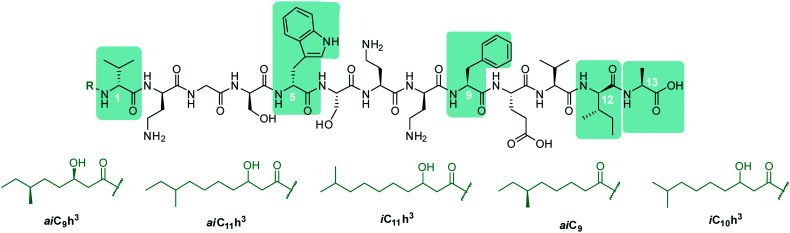							
Compound number	Tridecaptin variant	Variable residues					Lipid tail (R)^a^
		1	5	9	12	13	
**1** ^17,18^	A_1_ (A_α_)	d-Val	d-Trp	l-Phe	d-*a*Ile	l-Ala	*ai*C_9_h^3^, *ai*C_11_h^3^, iC_11_h^3^^b^
**2** ^18^	A_2_ (A_β_)	d-Val	d-Trp	l-Phe	d-Val	l-Ala	*ai*C_9_h^3^
**3** ^19^	A_3_	d-Val	d-Trp	l-Phe	d-*a*Ile	l-Ala	*ai*C_11_h^3^, iC_11_h^3^
**4** ^19^	A_4_	d-Val	d-Phe	l-Phe	d-*a*Ile	l-Ala	*ai*C_11_h^3^, iC_11_h^3^
**5** ^18^	B_α_	Gly	d-Trp	l-Ile	d-*a*Ile	l-Ser	*ai*C_9_
**6** ^18^	B_β_	Gly	d-Trp	l-Ile	dVal	l-Ser	*ai*C_9_
**7** ^18^	B_γ_	Gly	d-Trp	l-Val	d-*a*Ile	l-Ser	*ai*C_9_
**8** ^18^	B_δ_	Gly	d-Trp	l-Val	d-Val	l-Ser	*ai*C_9_
**9** ^18^	C_α1_	d-Val	d-Trp	l-Phe	d-Val	l-Ser	*ai*C_11_h^3^
**10** ^18^	C_α2_	d-Val	d-Trp	l-Phe	d-Val	l-Ser	iC_10_h^3^
**11** ^18^	C_β1_	d-Val	d-Trp	l-Phe	d-*a*Ile	l-Ser	*ai*C_11_h^3^

a Only stereochemistry of *ai*C_9_h^3^ and *ai*C_9_ have been reported.

b The lipid tail assignment of TriA_1_ (**1**) will henceforth be taken as *ai*C_9_h^3^ in line with elucidations by Lohans *et al.*^17^ Lipid tail nomenclature; *ai* = *anteiso*, i = iso. Uncommon amino acids native to tridecaptin; d-Dab (d-diaminobutyric acid), d-*a*Ile (d-*allo*-isoleucine).

In recent years, the tridecaptin class became a focus of research for the Vederas group. In their first report, they showed that the antibacterial agent produced by *Paenibacillus polymyxa* NRRL B-30509 with activity towards *Campylobacter jejuni* was actually TriA_1_, rather than a previously reported bacteriocin (SRCAM 602).^19^ The two most prevalent variants were tridecaptin A_3_ (**3**) and A_4_ (**4**) for which two possible lipid tails were identified as 3-hydroxy-8-methyldecanoyl (*ai*C_11_h^3^) and 3-hydroxy-9-methyldecanoyl (iC_11_h^3^) (Table 1), the former being the major lipid component. Having elucidated amino acid and lipid tail discrepancies within the TriA class of AMP, Lohans *et al.* subsequently recommended that the original naming system be replaced with a more rudimentary numbering system (A_α_ was henceforth to be known as A_1_, A_β_ as A_2_, *etc.*) to now encompass the tridecaptin A class.

After the initial resurgence of the tridecaptins, many new studies have been performed, including on their structural elucidation and/or biosynthesis,^17,22–24^ structure–activity relationships (SAR),^25–29^ mechanism of action,^30^ synergy with and conjugation to other antibiotics^31,32^ and as selective Gram-negative-staining agents.^33^

## 2. Resurgence of the tridecaptins: establishing a structure–activity relationship

**Fig. 1:**
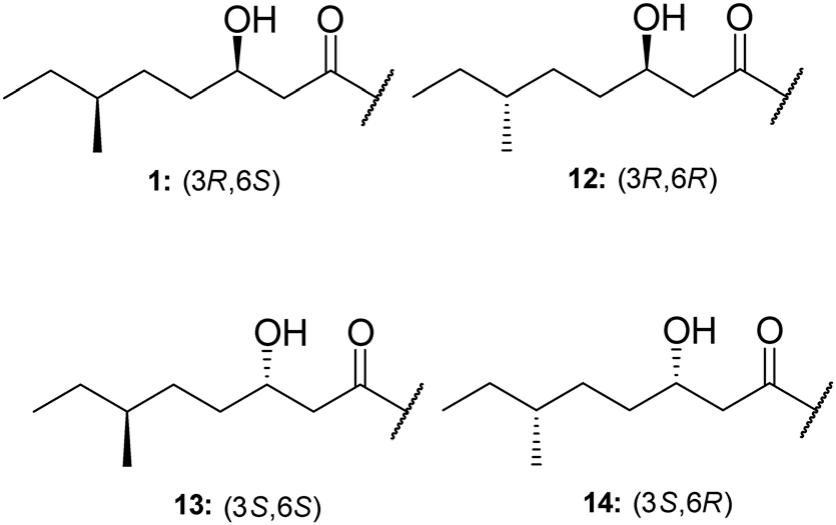
Chiral lipid tail variants of TriA_1_. A methodical approach was employed to assist in the complete stereochemical assignment of TriA_1_. Both HPLC and NMR were used to definitively ascertain the lipid tail stereochemistry as 3*R*,6*S*.

TriA_1_, (**1**) is a NRAP with robust bactericidal activity against Gram-negative bacteria.^26^ The tridecaptins belong to the linear cationic family of antimicrobial peptides; they possess 13 amino acids, including non-proteinogenic residues, and have a chiral lipid tail. Unfortunately, the need to incorporate a chiral moiety during peptide synthesis significantly increases their cost of synthesis (as well as adding several solution-phase synthesis steps to obtain the chiral lipid). In the first complete structural elucidation of a tridecaptin analogue, the Vederas group showed that TriA_1_ contained a (3*R*,6*S*)-3-hydroxy-6-methyloctanoyl lipid tail.^17^ This was achieved by chemically synthesizing the four possible lipid variants of TriA_1_ ((3*R*,6*S*) (**1**); (3*R*,6*R*) (**12**), (3*S*,6*S*) (**13**), and (3*S*,6*R*) (**14**)) (Fig. 1) and comparing these to the natural peptide using high-performance liquid chromatography (HPLC) and nuclear magnetic resonance (NMR) experiments. Interestingly, the chirality of the lipid tail did not have a significant impact on antimicrobial activity.

**Fig. 2:**
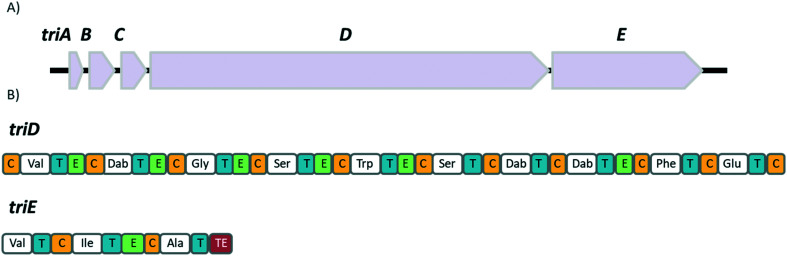
A) Biosynthetic gene cluster of TriA_1_ (from *P. terrae* NRRL B-30644) containing a predicted thioesterase (*triA*), two putative ABC transporter proteins (*triB* and *triC*) and NRPSs, *triD* and *triE*. B) Predicted domains of the NRPSs, *triD and triE*. Condensation domains (C) are depicted in gold, adenylation domains in white, thiolation domains (T) in blue, epimerase domains (E) in mint and the thioesterase domain (TE) in red.

In the same study, the Vederas group also sequenced the producer strain (*Paenibacillus terrae* NRRL B-30644) and identified the biosynthetic gene cluster (BGC) responsible for the production of the tridecaptins (Fig. 2), which contains a predicted thioesterase (*triA*), two putative ABC (ATP-binding cassette) transporter proteins (*triB* and *triC*) and two non-ribosomal peptide synthetases (*triD* and *triE*).

**Fig. 3:**
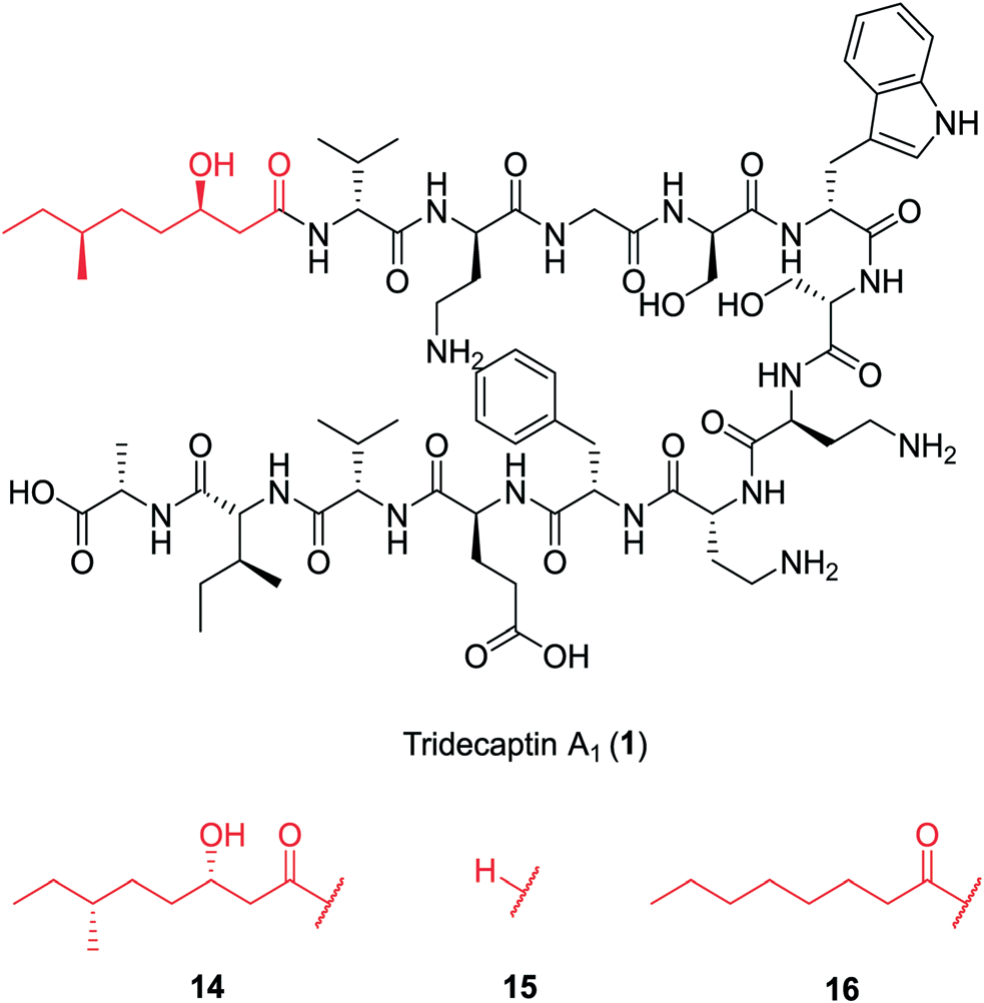
Variants of tridecaptin A_1_. The structures of natural tridecaptin A_1_ (**1**) and synthetic lipid tail analogues (**14–16**) are shown.

In a follow-up study, Cochrane *et al.* synthesized a library of TriA_1_ lipid-chain analogues (**16–32**).^26^ The major finding from this work was that that chirality of the lipid chain was not essential for activity (although it was noted that inversion of both stereochemical centres along the lipid tail gave a four-fold decrease in activity). The lipid tail on the tridecaptins is essential for activity, as truncated analogue H-TriA_1_ (**15**) (Fig. 3) displayed a significant reduction in antimicrobial activity against *Escherichia coli* (MIC = 100 μg mL^−1^) compared to the native peptide (**1**) (MIC = 3.13 μg mL^−1^). From the range of analogues tested, Oct-TriA_1_ (**16**) consistently gave the most potent activity against all strains tested. Various lipid tail lengths were reasonably well tolerated (C_4–12_, **18–24**) and only showed two- to four-fold decreases in activity against the model Gram-negative strain, *E. coli*, when compared to the natural peptide (**1**).^26^ However, when longer lipid chains were used (C_14/16_, **25–26**), activity was abolished across most strains (MIC >100 μg mL^−1^), likely due to the poor solubility of analogues. The necessity of a lipid tail for antimicrobial activity, and indeed the correlation to chain length, aligns with the behaviour of lipopeptides such as TriA_1_ to act as cell membrane disrupters. Indeed, it was found that the truncated derivative, H-TriA_1_ (**15**), exhibits reduced cytotoxic effects when compared to the native peptide, owing to its ability to sensitize the bacterial membrane, rather than disrupt it.^26,31^ The discovery that the synthetic analogue, Oct-TriA_1_ (**16**), retained all properties of the natural peptide, provided a significantly cheaper and more efficient method to access tridecaptin analogues. Oct-TriA_1_ was also found to exhibit strong activity against multidrug resistant *Klebsiella and Enterobacter* strains.^26^

Despite its lack of antimicrobial activity, the unacylated analogue H-TriA_1_ (**15**) has been shown to work synergistically with a range of antibiotics, where it acts as a strong outer-membrane sensitizer of Gram-negative bacteria.^31^ In the first study on the synergistic effects of H-TriA_1_ with other antibiotics, it was found that sub-MIC concentrations of H-TriA_1_ reduced the MIC of clinically relevant antibiotics rifampicin and vancomycin against *E. coli* by up to 512- and 64-fold, respectively. H-TriA_1_ (**15**) can also act as a sensitizer of MDR *Klebsiella pneumoniae*, improving susceptibility to antibiotic treatments by up to 512-fold. Recently, a collaborative effort between the Cochrane, Payne, Martin and Vederas groups, and the Ferring Research Institute, showed that H-TriA_1_ significantly improves the antimicrobial activity of teixobactin analogues to include Gram-negative strains.^34^ Perhaps the most significant observation was a 125-fold decrease in MIC of native teixobactin against *Salmonella enterica* when co-administered with H-TriA_1_ at 12.5 μg mL^−1^.

While partial membrane lysis was ruled out as a potential explanation for the synergistic effects of H-TriA_1_ (**15**),^31^ the similarity of its circular dichroism (CD) spectra in the presence of large unilamellar vesicles (LUVs) as model membranes, to that of the synthetic analogue, Oct-TriA_1_ (**16**),^25^ suggests that formation of a stable secondary structure on the OM may facilitate sensitization of Gram-negative bacteria. In a later paper describing the mechanism of action of the tridecaptins, it was shown by isothermal titration calorimetry (ITC) that these peptides bind to lipopolysaccharide (LPS), a glycopolymer found on the surface of the outer membrane.^30^ Therefore, it is likely that H-TriA_1_ binding to LPS is the mechanism of sensitization, which is how the related polymyxin B nonapeptide (PMBN) sensitizes Gram-negative bacteria.^35^

**Fig. 4:**
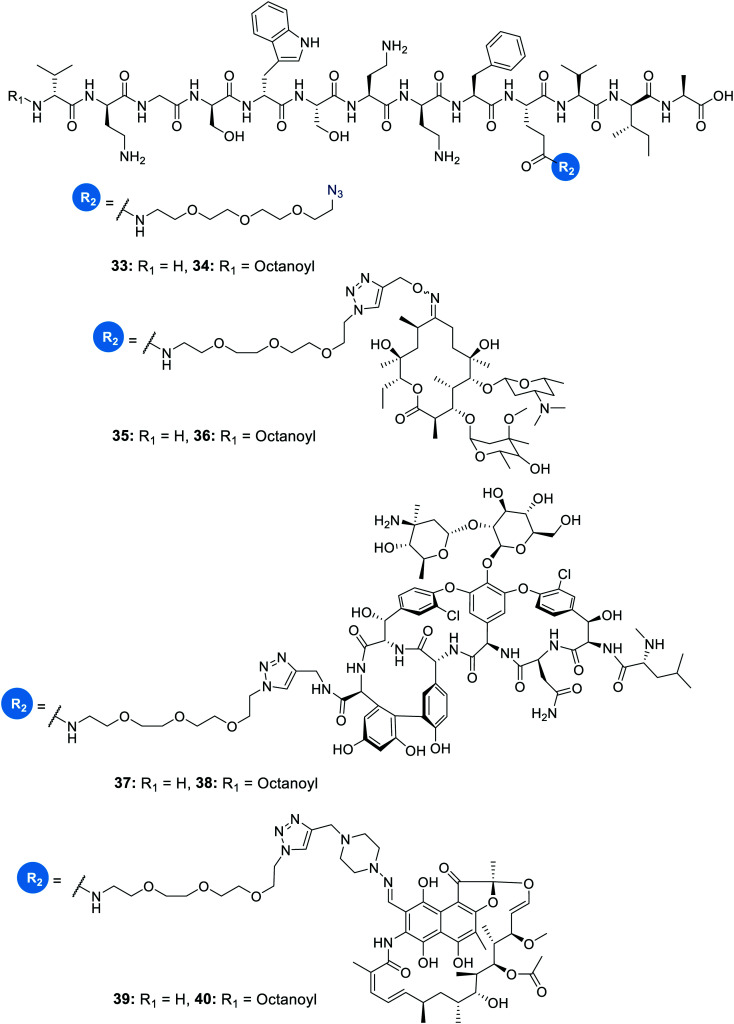
Azidopeptides and covalently linked tridecaptin–antibiotic conjugates. The structures of azidopeptides, H-TriA_1_(Glu10-PEG_3_N_3_) (**33**) and Oct-TriA_1_(Glu10-PEG_3_N_3_) (**34**), are shown alongside tridecaptin–antibiotic conjugates, H-TriA_1_-Eryc (**35**), Oct-TriA_1_-Eryc (**36**), H-TriA_1_-Van (**37**), Oct-TriA_1_-Van (**38**), H-TriA_1_-Rif (**39**) and Oct-TriA_1_-Rif (**40**).

In another study on the synergistic effects of the tridecaptins with existing antibiotics, H-TriA_1_ and Oct-TriA_1_ were covalently linked to the antibiotics rifampicin, vancomycin and erythromycin.^32^ Vancomycin and erythromycin are inactive against Gram-negative bacteria due to their inability to cross the outer membrane, so it was hoped that conjugation to the tridecaptins could allow their passage through this barrier. Copper-assisted azide-alkyne cycloaddition (CuAAC) was the method used for conjugation and Glu10 was identified as an ideal site to install an azide group on H-TriA_1_ and Oct-TriA_1_. Azidopeptides H-TriA_1_(Glu10-PEG_3_N_3_) (**33**) and Oct-TriA_1_(Glu10-PEG_3_N_3_) (**34**) (Fig. 4) both retained their respective antimicrobial and synergistic activities. Conjugation to an erythromycin alkyne, vancomycin alkyne or rifampicin alkyne yielded tridecaptin antibiotic conjugates **35–40** in moderate – good yields (Fig. 4). When screened for *in vitro* activity against *E. coli*, *K. pneumoniae* and *A. baumannii* strains, the tridecaptin-antibiotic conjugates were much less effective than a synergistic mixture of the individual components. However, when tested in a mouse-infection model, it was found that the erythromycin–H-TriA_1_ conjugate (**35**) was more effective at promoting mouse survival than the synergistic combination. In this *in vivo* model, it was found that the synergistic combinations were not particularly effective, although this was just a single study with a small number of animals.

**Fig. 5:**
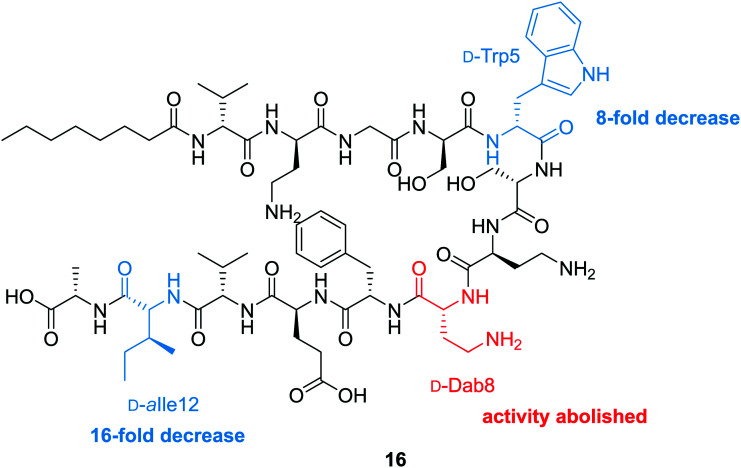
Synthetic Oct-TriA_1_ analogue (**16**) with key residues highlighted. Substitution of these d-amino acid residues, Trp5, Dab8 and *a*Ile12, resulted in the observed reductions in activity (relative to Oct-TriA_1_) against *E. coli* ATCC 25922. The most significant reduction in activity was associated with replacement of the d-Dab8 residue, suggesting it plays an intrinsic role in antimicrobial activity.

To probe the mechanism of action of the tridecaptins, the Vederas lab performed an alanine scan on Oct-TriA_1_ (**16**).^25^ The antimicrobial activity of all alanine scan analogues (**41–52**) was determined and their secondary structure in the presence of model membranes was analyzed using CD spectroscopy. Residue substitutions that proved most detrimental to antimicrobial activity were d-Dab8, d-*allo*-Ile12 and d-Trp5 (Fig. 5). In particular, substitution of the cationic residue d-Dab8 completely abolished antimicrobial activity, suggesting this site plays an important role in the mechanism of action of the peptide.^25^ Given that both d-*allo*-Ile12 and d-Trp5 are hydrophobic residues, it was speculated that their substitution causes a significant reduction in the hydrophobicity of Oct-TriA_1_ and impacts formation of a stable secondary structure. CD spectroscopy was employed to assess how alterations to the peptide sequence altered its secondary folding. 50 nm phospholipid LUVs were used to mimic the bacterial membrane environment.^25^

The CD spectra of each alanine scan analogue was then recorded to assess the effects of amino acid substitution on formation of a stable secondary structure. It was found that reduction of the intensity of the negative band at 194 nm correlated with a loss in antimicrobial activity. Substitution of essential residues d-Dab8, d-*allo*-Ile12 and d-Trp5 significantly reduced activity and disrupted native peptide folding, verifying that a sequence-activity relationship exists, that when altered, impedes efficient peptide folding.^25^ In this study the authors also showed that the enantiomeric form of Oct-TriA_1_, *Ent*-Oct-TriA_1_ (**53**) was ∼four-fold less active, suggesting a chiral receptor may be involved in the mechanism of action.

In 2016, Cochrane, Findlay and Vederas finally identified a plausible mechanism of action for how the tridecaptins kill Gram-negative bacteria. Bactericidal growth kinetics and membrane lysis experiments showed that TriA_1_ rapidly kills bacteria, consistent with a membrane-targeting antimicrobial. However, follow-up membrane depolarization and membrane lysis assays showed that TriA_1_ does not significantly disrupt the cell membrane. This result was corroborated in a subsequent study by Sean Brady and co-workers as part of their work on synthetic-NRPs.^36^ Subsequent experiments suggested that TriA_1_ disrupts the proton-motive force *via* the formation of proton-specific pores within the membrane of Gram-negative bacteria, altering cytoplasmic pH and thus leading to bacterial cell death, likely due to prevention of ATP synthesis.^30^ With a mode of action identified, Cochrane *et al.* then looked for possible receptors to which TriA_1_ binds to to cross the outer membrane and interact with the inner membrane.

**Fig. 6:**
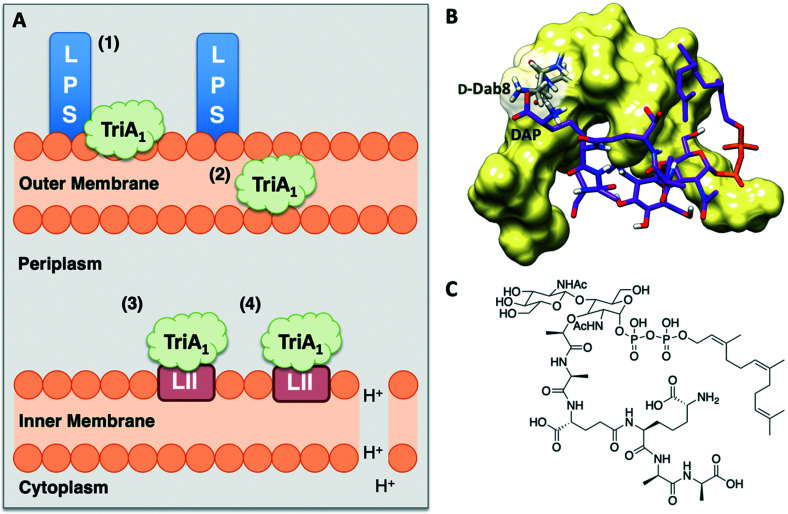
A) Summary of TriA_1_ mechanism of action.^30^ TriA_1_ interacts with LPS on the surface of the outer membrane (1). It then crosses the outer membrane (2) and enters the periplasm. TriA_1_ then binds to Gram-negative lipid II on the outer leaflet of the inner membrane (3) and disrupts the proton motive force (4). B) Model of TriA_1_-lipid II complex. Figure reproduced from deposited solution NMR structure of Oct-TriA_1_ in DPC micelles containing Gram-negative lipid II structure (PDB: 2N5Y) (obtained from Dr Stephen Cochrane). Lipid II is shown as a stick figure, with backbone in purple, and Oct-TriA_1_ shown as a surface representation. The interaction between d-Dab8 and DAP is highlighted, with the peptide visible as a stick figure with backbone in grey. C) Structure of synthetic Gram-negative lipid II analogue. In this study a synthetic Gram-negative lipid II analogue containing a shorter prenyl chain was used as this modification improved solubility for NMR studies.

Isothermal titration calorimetry showed that TriA_1_ (**1**), Oct-TriA_1_ (**16**), the enantiomeric form of TriA_1_ (*Ent*-TriA_1_ (**53**)) and H-TriA_1_ (**15**) all bind to LPS with similar affinities. This suggested that TriA_1_ crosses the outer membrane by first binding to LPS. Further ITC experiments showed a remarkable selectivity of TriA_1_ for the Gram-negative version (contains diaminopimelic acid (DAP) rather than lysine) of the peptidoglycan precursor lipid II. Incubating TriA_1_ with Gram-negative lipid II before antimicrobial testing sequestered activity through complex formation and an intricate *in vitro* assay showed that LUVs doped with Gram-negative lipid II resulted in much faster proton leakage than undoped or Gram-positive lipid II doped vesicles. A solution NMR structure of Oct-TriA_1_ in the presence of Gram-negative lipid II and dodecylphosphocholine (DPC) micelles was determined and docking experiments used to produce a model of this interaction. The resulting model (Fig. 6) showed a key interaction between TriA_1_d-Dab8, the only truly essential residue, and lipid II DAP, the major structural difference between Gram-negative and Gram-positive lipid II.^30^ This may be why the tridecaptins specifically kill Gram-negative-bacteria. Taken together, this study provided a plausible mechanism of action for TriA_1_, by which it binds to LPS on the outer membrane, enters the periplasm, binds to lipid II on the outer leaflet of the inner membrane and disrupts the proton-motive force.

## 3. How robust are tridecaptins towards antimicrobial resistance?

**Fig. 7:**
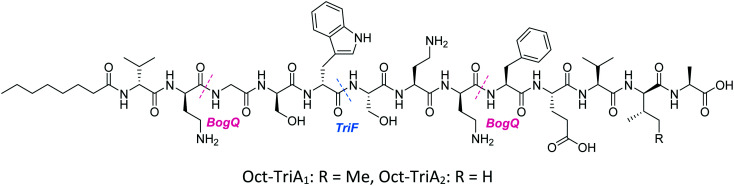
Cleavage sites on Oct-TriA variants of BogQ and TriF d-peptidase enzymes. BogQ hydrolyses the amide bond at the C-terminus of cationic d-Dab residues whilst TriF voids the peptide of antimicrobial activity by cleaving at the C-terminus of d-Trp5.

Initial assessment of bacterial resistance towards the tridecaptins yielded positive results; a resistance development study in which *E. coli* cells were exposed to sub-MIC concentrations of Oct-TriA_1_ (**16**) for one month saw no resistance development but an eightfold reduction in activity was observed against the ciprofloxacin control.^30^ The lack of resistance development against the tridecaptins may be associated with the importance of their lipid II target in peptidoglycan biosynthesis. As this precursor is essential for formation of the bacterial cell wall, modification of this intermediate is unlikely due to potential deleterious effects downstream. Therefore, development of a ‘self-destructive’ resistance mechanism is unlikely. Unfortunately, a self-protection mechanism in tridecaptin-producing strains was recently identified that could have implications for their use as therapeutic agents.^37^

d-Stereospecific resistance peptidase (DRP) enzymes, BogQ and TriF (from *Brevibacillus laterosporus* and *P. polymyxa* strains respectively), were heterologously expressed in *E. coli* and it was shown that the conserved catalytic region in each (Ser-XX-Lys and Tyr-XX) was the basis of peptidase activity.^37^ Both DRPs hydrolyse Oct-TriA_1_ (**16**) at the C-terminus of d-amino acid residues in the peptide chain, with BogQ showing specificity for d-cationic amino acids d-Dab2 and d-Dab8, and TriF showing selectivity for d-aromatic amino acid d-Trp5 (Fig. 7). Hydrolysis by either enzyme renders the peptide inactive. It is speculated that bacteria produce both BogQ and TriF d-stereospecific resistance peptidases as a self-protection strategy to evade cell death by their own antimicrobial metabolites. Worryingly, this level of inherent resistance has the potential to mobilize into pathogenic bacteria *via* horizontal gene transfer, potentially hindering the development of tridecaptins as novel therapeutic agents.^37^

**Fig. 8:**
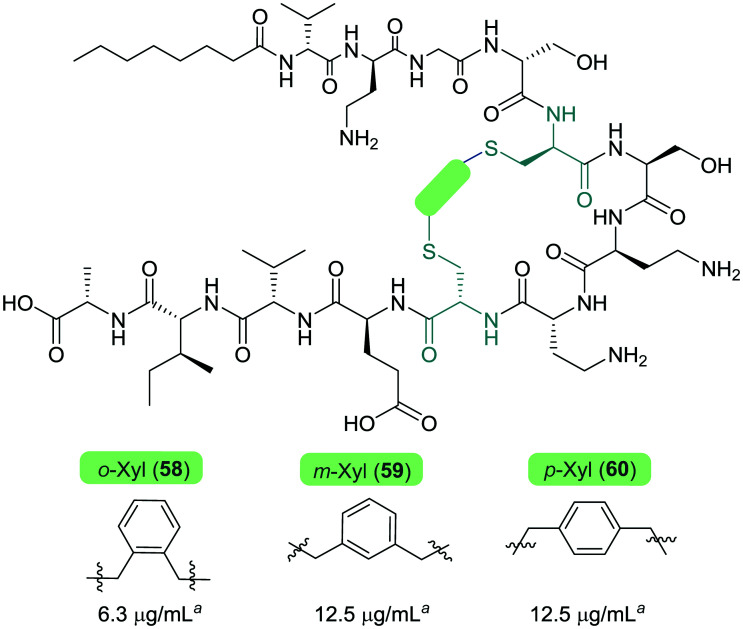
Cyclized Oct-TriA_1_ analogues (**58–60**), in which the characteristic π-stacking interaction has been replaced by regiospecific xylyl linkers. ^*a*^ MIC values against *A. baumannii* NCTC 13304.

The Cochrane lab recently identified modifications that improve the stability of the tridecaptins to d-peptidase hydrolysis.^27–29^ Analysis of the NMR solution structure of Oct-TriA_1_ in DPC micelles containing Gram-negative lipid II suggests that a π-stacking interaction between d-Trp5 and l-Phe9 may be important to maintain the looped structure of the peptide.^30^ TriF cleaves tridecaptins at d-Trp5, so the authors postulated that replacement of this π-stacking interaction with a covalent linkage to form a cyclic peptide could prevent TriF hydrolysis.^27^ A small library of peptides were synthesized in this study (**54–61**), including macrocyclic peptides wherein a d-Cys5 and l-Cys9 residue are crosslinked with various linkers. Macrocyclic peptides **58–60** (Fig. 8) retained strong activity against *E. coli* and even displayed comparable potency against the highly problematic *A. baumannii* strain,^27^ which the WHO currently rank as the no. one pathogen necessitating imminent novel therapeutic treatment.^10^ As anticipated, Xyl-linked analogues **58–60** were resistant to hydrolysis by the DRP enzyme, TriF. After a 12 h incubation period with the soluble periplasmic peptidase domain of TriF, TriF_pep_, UPLC-MS analysis revealed that while Oct-TriA_1_ (**16**) was hydrolysed, peptides **57–60** were stable to enzymatic degradation. The strategic design of these novel covalently linked macrocycles presents a new class of cyclic tridecaptin analogue with strong antimicrobial activity and an acquired ability to withstand d-peptidase mediated hydrolysis by TriF.

Conservative amino acid substitutions have also been applied to both Oct-TriA_1_ (**16**) and Oct-TriA_2_ (**62**) variants to mitigate hydrolysis by BogQ and TriF enzymes and reduce cost of synthesis. Initially, Dab at position 7 and/or d-Dab at positions 2 and 8 were replaced with the longer chain cationic amino acids (with the same l/d-configuration), lysine (Lys) or ornithine (Orn), yielding peptide library **63–68**.^28^ It was postulated that these moderate alterations would not be deleterious to activity while producing analogues that could be synthesized at a considerably reduced cost. Oct-TriA_1_ (**16**) was found to be two- to four-fold more active than its variant Oct-TriA_2_ (**62**), which has d-Val12 in place of d-*allo*-Ile12. However, synthesis of the latter is much more cost-effective, as Fmoc-d-Val is much cheaper than Fmoc-d-*allo*-Ile.‡‡Fmoc-d-*allo*-Ile-OH: £154 per g, Fmoc-d-Val-OH: £19.60 per g (prices from Alfa Aesar catalogue – accurate as of 27/11/20). Synthetic analogues substituted at positions 2 and 7 with Lys or Orn (of the correct l/d-configuration) showed comparable activity to one another, but significantly reduced activity against most strains when compared to control peptide Oct-TriA_1_ (**16**). Disubstituted analogues Oct-TriA_1_ (d-Orn2, Orn7) (**63**) and Oct-TriA_1_ (d-Lys2, Lys7) and (**64**) were found to be eight-fold less active against the carbapenemase-producing strain, *Enterobacter cloacae*, yet showed enhanced activity against *Pseudomonas pseudoalcaligenes*.^28^ Although switching native Dab residues for Lys/Orn seemed to hinder activity, preservation of antimicrobial activity led the authors to focus on triply substituted analogues. Analogues **65–68** would incorporate substitutions at positions 2, 7 and 8 to assess the impact, if any, on switching out the essential d-Dab8 residue, that is key to lipid II binding,^30^ for longer chain cationic amino acids. The most promising analogues to note were Oct-TriA_1_ (d-Orn2,8, Orn7) (**65**), Oct-TriA_1_ (d-Lys2,8, Lys7) (**66**) and Oct-TriA_2_ (d-Orn2,8, Orn7) (**67**); each is cheaper to synthesize than Oct-TriA_1_ (**16**) and retained antimicrobial activity relative to its unsubstituted precursor. Peptides **66** and **67** even showed enhanced activity against strains of the critical priority pathogen, *A. baumannii*, when compared to their relevant unsubstituted predecessor. It was found that replacing d-Dab8 with d-Lys was deleterious to activity, perhaps explained by the two-methylene increase in chain length when the native Dab residue is replaced with Lys rather than Orn. As the site in question is involved in lipid II binding, it would be reasonable to suggest that a considerable lengthening of the side chain would impede its bactericidal action, however binding studies are necessary to verify this assumption.

Despite the encouraging discovery of Oct-TriA_2_ (d-Orn2,8, Orn7) (**67**), with its strong antimicrobial activity and reduced synthetic cost, this peptide (and other analogues **16** & **62–68**) were susceptible to hydrolytic cleavage by the d-peptidase BogQ (Table 2). When incubated with BogQ for 1 h, peptide **67** suffered >90% hydrolysis.^29^ The same pattern of degradation was also observed for xylyl-linked cyclic TriA_1_ analogues (**58–60**), further emphasising the ever-growing threat bacteria pose to the development of new antibiotic candidates.

**Table 2:** Synthetic tridecaptin analogues intended to circumvent d-peptidase mediated hydrolysis

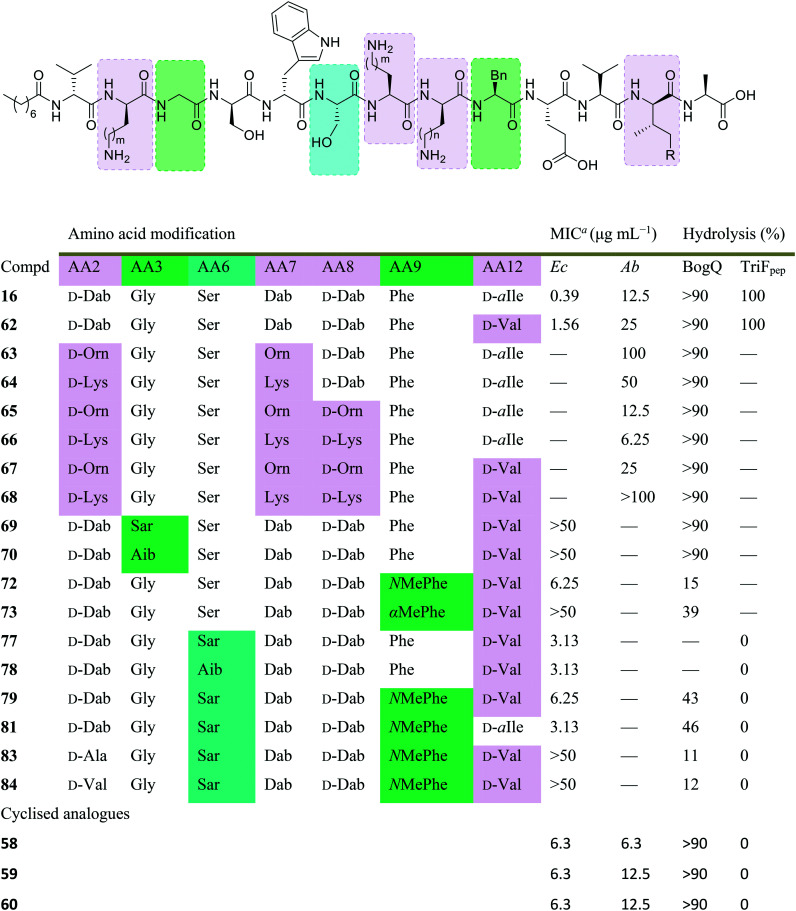

a MIC: minimum inhibitory concentration. Strains referenced are *E. coli* NCTC 12241 (Ec) and *A. baumannii* (Ab) (peptides **58–60**: NCTC 13304, **16** & **62–68**: environmental strain).

In a follow-up study, the Cochrane lab looked for alternative modifications that could be made to a linear tridecaptin scaffold that improved their resistance to TriF and/or BogQ hydrolysis.^29^ To this end, a series of common modifications that improve peptide stability to peptidases were explored, including *N*-methylation, α-methylation and inversion of stereochemistry at hydrolysis sites. Residues 3, 6 and 9, residing at the C-termini of BogQ and TriF cleavage sites (Fig. 7), were chosen for modification, with the ultimate goal to sterically obstruct all cleavage sites by developing a triply substituted peptide that was completely stable to BogQ and TriF-mediated hydrolysis. As such, a library of new Oct-TriA_2_ analogues (**69–84**) were synthesized and tested. Replacement of Gly3 with bulkier *N*- and α-methylated residues, sarcosine (Sar, **69**) or 2-aminoisobutyric acid (Aib, **70**) was detrimental to antimicrobial activity at the upper limits tested (MIC >50 μg mL^−1^). Adding a methyl group at position 3 significantly reduces the rotational freedom of the archetypal peptide backbone^29^ and so was inferred to disturb the looped structure that Oct-TriA_1_ (**16**) forms when in contact with the lipid II target.^30^ Monosubstituted analogues bearing these alterations (**69**, **70**) were also rapidly hydrolysed by BogQ (>90%) (Table 2). Perturbation of BogQ hydrolysis at d-Dab8 was achieved *via* replacement of Phe9 with its *N*-methylated variant, *N*MePhe (**72**). Enzymatic cleavage fell considerably to 15% and antimicrobial activity was retained (MIC = 6.25 μg mL^−1^). Despite boosting stability against BogQ-mediated hydrolysis (39%), substitution with the α-methylated residue, αMePhe (**73**), abolished activity at the limits tested. Knowing how such sequence alterations at positions 3 and 9 impacted antimicrobial activity, the focus was then directed to position 6 and how similar changes could prevent TriF hydrolysis. TriF cleaves Oct-TriA_1_ (**16**) at d-Trp5, therefore, Sar or Aib was placed at position 6 to increase steric bulk of the native l-Ser6 residue. Both analogues (**77**, **78**) retained strong antimicrobial activity (MIC = 3.13 μg mL^−1^) relative to the parent peptide, Oct-TriA_2_ (**62**) and no TriF-mediated hydrolysis was observed. Analogues **77** and **78** are more active than the novel cyclic Xyl-linked peptides (**58–60**),^27^ showing that a simple amino acid substitution on the linear scaffold is a more efficient strategy to yield highly active peptides that are completely stable towards TriF-mediated hydrolysis.^29^

After establishing which single amino acid changes improved BogQ and TriF stability, multi-substituted analogues were then synthesized in an attempt overcome d-peptidase hydrolysis at all sites. Combining the most active single mutants produced disubstituted analogue Oct-TriA_2_ (Sar6, *N*MePhe9) (**79**), which retained strong antimicrobial activity (MIC = 6.25 μg mL^−1^), noticeably reduced BogQ-mediated hydrolysis (43%) and was completely resistant to TriF cleavage.^29^ Similar findings were obtained when d-Val12 was replaced with d-*allo*-Ile, yielding the more expensive Oct-TriA_1_ (Sar6, *N*MePhe9) (**81**). It too was completely stable to TriF and experienced comparative BogQ hydrolysis (46%) but was two-fold more active (MIC = 3.13 μg mL^−1^) than the cheaper counterpart (**79**). The authors also explored replacing the cationic d-Dab2 residue with hydrophobic amino acids d-Ala (**83**) or d-Val (**84**), and although the resulting analogues displayed high levels of resistance to both BogQ and TriF, they lacked antimicrobial activity at the upper limits tested.

Haemolytic assays were conducted on the propitious analogues Oct-TriA_2_ (*N*MePhe9) (**72**), Oct-TriA_2_ (Sar6) (**77**) and Oct-TriA_2_ (Sar6, *N*MePhe9) (**79**). Each exhibited low levels of haemolytic activity (30–60%) when compared to the parent peptide Oct-TriA_2_ (**62**, 53%).^29^ Additional spot-on-lawn assays with *E. coli* NCTC 12241, in the presence of lipid II, confirmed retention of their receptor-mediated mechanism of action. Discovery of these tridecaptin-inspired analogues demonstrates an ability to overcome d-peptidase resistance with cost-effective, yet highly active linear tridecaptin peptides, that retain their intricate mechanism of action and do not show signs of haemolytic toxicity.

## 4. Extended applications of tridecaptin and discovery of other native variants

**Fig. 9:**
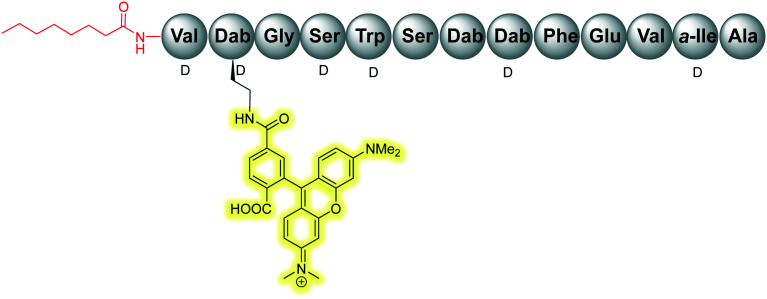
TriA-TAMRA probe (**85**) used for differential staining of Gram-negative bacteria.

The scope of the tridecaptins has gone beyond their use as antimicrobial peptides, as they have recently found application as a Gram-negative-specific fluorescent probe.^33^ Synthesis of a single-structure probe that selectively identifies Gram-negative bacteria helps overcome discrepancies inherent to the traditional Gram-staining technique,^38^ and indeed other chemical probes. The fluorophore tag, tetramethylrhodamine (TAMRA) was covalently linked to Oct-TriA_1_ (**16**) *via* the side chain of d-Dab2 to yield Oct-TriA_1_(d-Dab2-TAMRA) (**85**) (Fig. 9). This tridecaptin-conjugated probe (**85**) not only showed superior labelling of fixed bacteria compared to a polymyxin B based probe, which lends itself to practical applications, but also has capacity to selectively label Gram-negative bacteria in the midst of complex samples.^33^ Coupled with ease of synthesis, it is suggested that such a probe has the capability to differentially stain Gram-negative bacteria with a significant degree of accuracy that circumvents the intrinsic flaws of traditional Gram-staining.

**Fig. 10:**
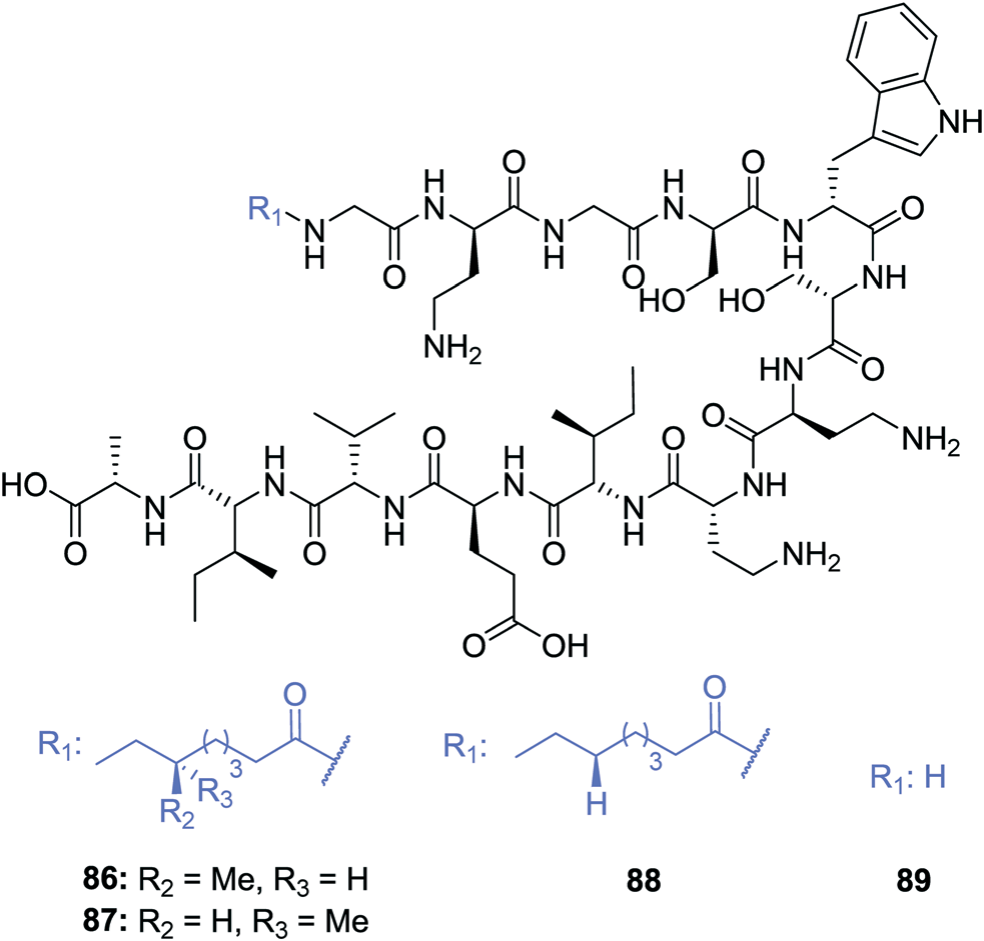
Structures of tridecaptin B variants: (6*S*)-TriB_1_ (**86**), (6*R*)-TriB_1_ (**87**), Oct-TriB_1_ (**88**) and H-TriB_1_ (**89**).

Other natural tridecaptin variants have also been reported, namely TriB_1_ (**86**) and TriM_1_ (**90**). TriB_1_ (**86**), isolated from a mixture of antimicrobials purified from an overnight culture of *P. polymyxa* NRRL B-30507, was fully characterized by Cochrane *et al.*^22^ C_18_-HPLC purification of this culture extract yielded three active fractions, two of which were determined to be polymyxin B_1_ and B_2_. However, the low level of anti-*Campylobacter jejuni* activity of the polymyxins^39^ suggested another metabolite must be responsible for this antimicrobial activity. Based on its analytical similarity to TriA_1_ (**1**) the third antimicrobial was identified as TriB_1_ (**86**) which differs slightly to TriA_1_, in that it contains a (6*S*)-methyloctanoic acid tail, glycine in place of d-Val1 and isoleucine replaces l-Phe9 (Fig. 10).^22^

**Fig. 11:**
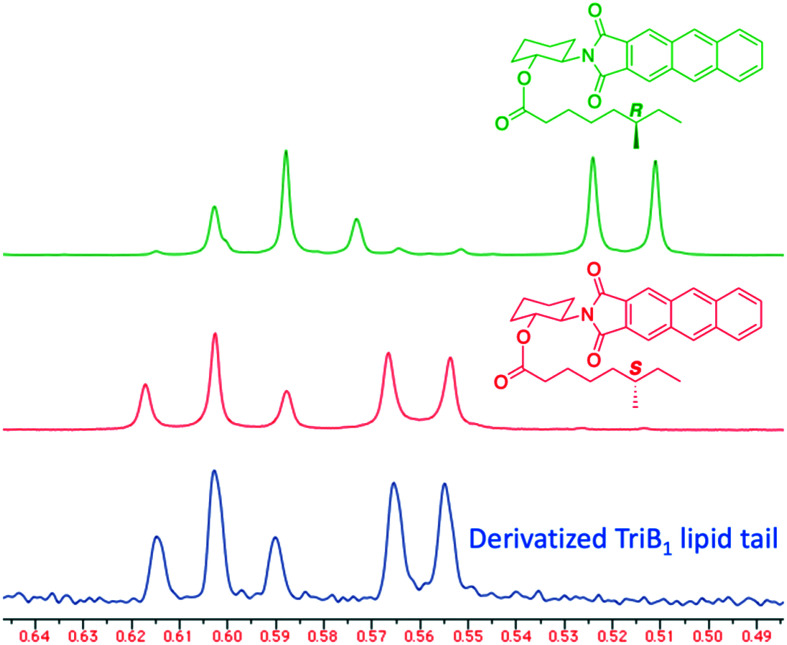
Elucidation of TriB_1_ lipid chain stereochemistry. (6*R*)-Methyl derivative (**90**, green) and (6*S*)-methyl derivative (**91**, red) were prepared by chemical synthesis. The lipid tail from TriB_1_ was obtained by hydrolysis and derivatized with the same anthracenyl unit (blue). ^1^H-NMR analysis allowed the chirality of the lipid tail to be determined as *S*. *X*-Axis units are parts per million (ppm).

Of particular note in this study is how the authors identified the stereochemical configuration of the TriB_1_ lipid tail. Unlike TriA_1_, it was not possible to distinguish between (6*S*)-methyloctanoyl-TriB_1_ (**86**) and (6*R*)-methyloctanoyl-TriB_1_ (**87**) (which were obtained by chemical synthesis) using HPLC or NMR. To identify the stereochemistry, the authors utilized the Ohrui–Akasaka method,^40^ where enantiomerically pure chiral lipids were converted into anthracenyl derivatives which, due to the different ring current experienced by an *R*- or *S*-methyl group, can be distinguished using ^1^H-NMR. The lipid tail was hydrolyzed from natural TriB_1_ and derivatized in the same manner, and comparison with the synthetic standards unambiguously showed that the 6-methyl group had the *S*-configuration (Fig. 11).

Much like TriA_1_ (**1**), TriB_1_ (**86**) exhibits strong activity against various Gram-negative strains (MIC = 3.13–6.25 μg mL^−1^), including a strain of MDR *K. pneumoniae,* but shows negligible activity against Gram-positive pathogens (MIC >100 μg mL^−1^). Although more cost-effective, synthetic variant Oct-TriB_1_ (**88**) experienced a two- to four-fold reduction in antimicrobial activity. Further similarities were found between TriA_1_ and B_1_; much like H-TriA_1_ (**15**), H-TriB_1_ (**89**) is devoid of antimicrobial activity but acts as an effective sensitiser of the front-line antibiotic, rifampicin, increasing its efficacy against *E. coli* 64×.^22^ The biosynthetic origins of TriB_1_ (**86**) were investigated *via* elucidation of its associated BGC. A BGC analogous to that of TriA_1_ (**1**) was found that differed only in the selectivity of adenylation domains 1 and 9. Although these differences could not be attributed to the flexibility of amino acids 1 and 9 found in tridecaptins A and B, identification of diversifying NRPSs emphasises the ability of these modular enzymes to produce heterogenous antimicrobial peptides that could be utilised as potent antibiotic drug candidates.

Recently, TriM_1_ (**92**) (Table 3) was isolated from a mud bacterium culture, *Paenibacillus* sp. M-152, and is reported to display activity against colistin-resistant and MDR *Enterobacteriaceae* strains, and *K. pneumoniae*.^23^ Similar to the classification of TriB_1_ (**86**), TriM_1_ was identified and its structure assigned *via* extensive analytical and NMR work and its initial presence postulated due to observed activity against resistant strains that could not be credited to existing antibiotics. TriM_1_ (**92**) has a similar structure to TriB_1_, with the exception of two amino acid positions; l-Val11 is replaced with l-Ile and l-Ala13 is instead l-Ser. Although two- to four-fold less active against polymyxin-sensitive strains of *K. pneumoniae*, TriM_1_ displayed superior activity against extensively drug-resistant (XDR) strains (MIC = 2 μg mL^−1^).^23^ Much like the tridecaptin A and B variants, analogue TriM_1_ (**92**) showed no activity against Gram-positive bacteria at the upper limits tested (MIC >128 μg mL^−1^) and its biosynthetic origins were attributed to a BGC comparable to that obtained for TriA and B.^22^

**Table 3:** Structures of TriM variants and associated antimicrobial activity against Gram-negative bacteria

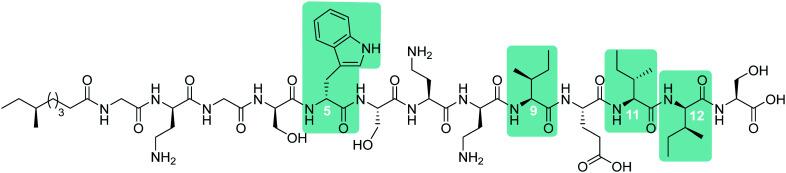							
Compound no.	Analogue	Variable residues				MIC (μg mL^−1^)	
		5	9	11	12	*K. pneumoniae AH-3 (Col-R)*	*S. marcescens MTCC 97*
**92**	M_1_	d-Trp	l-Ile	l-Ile	d-*a*Ile	4	8
**93**	M_2_	d-Trp	l-Ile	l-Val	d-Val	8	>16
**94**	M_5_	d-Phe	l-Ile	l-Ile	d-Val	16	8
**95**	M_6_	d-Trp	l-Ile	l-Ile	d-Val	4	16
**96**	M_7_	d-Trp	l-Val	l-Ile	d-*a*Ile	8	8
**97**	M_8_	d-Phe	l-Ile	l-Ile	d-*a*Ile	8	16
**98**	M_11_^a^	ND	l-Phe	l-Ile	d-*a*Ile	1	4

a Residues 1–7 could not be conclusively determined.

Time-kill assays of TriM_1_ at different concentrations confirmed it to be bactericidal in nature and exposure of *K. pneumoniae* to sublethal concentrations of TriM_1_ showed low-levels of resistance development. Mode-of-action studies aligned with data found for TriA_1_ (**1**)^30^ as it was found that TriM_1_ (**92**) disrupts the proton-motive force, likely blocking ATP synthesis.^23^ The susceptibility of polymyxin-resistant *K. pneumoniae* to TriM_1_ suggests that if the tridecaptins do indeed interact with LPS, as previously reported,^30^ it must be by a different mechanism than the polymyxins. Co-administration of TriM_1_ with subinhibitory concentrations of the OM-targeting antibiotic, colistin, reduced the MIC of TriM_1_ 16-fold, further emphasising the role tridecaptins can play in forming synergistic relationships with clinically relevant antibiotics. TriM_1_ (**92**) has also been shown to act synergistically against the highly problematic bacterium *A. baumannii* in combination therapy with the clinically relevant antibiotic, rifampicin.^41^ The natural peptide (**92**) was proven to act as a outer membrane potentiator, reducing the MIC of rifampicin against the *A. baumannii* GMCH05 strain by 256×, when combined with TriM_1_ (**92**) at a concentration of 8 μg mL^−1^. This example of combination therapy was also demonstrated to tolerate a lag administration of each drug, as well as significantly reducing the presence of persister cells post treatment, a timely find in the continued fight to develop effective treatments for problematic bacterial infections.

Finally, *in vitro* experiments of novel TriM_1_ (**92**) found a favourable toxicology profile; the peptide exhibited low haemolysis and cytotoxicity, and no nephrotoxicity was observed at the same dose at which colistin was lethal.^23^ These observations are consistent with all available toxicity data for natural and synthetic TriA and TriB analogues. Such findings support the potential for the future development of tridecaptins as antibiotic agents, owing to their potency for highly problematic Gram-negative bacteria and their favourable *in vivo* activity.

Following on from the discovery of a new class of tridecaptin peptides, a further ten M variants were isolated from the same M-152 strain, however only six were completely characterised (**92–97**; M_1–2_, M_5–8_) with M_11_ (**98**) partially assigned (Table 3).^24^ These novel variants are attributed to the same BGC as the forerunner, TriM_1_ (**92**), highlighting the diversity afforded by non-ribosomally synthesized peptides. Of the six variants characterised, amino acids at positions 5, 9, 11 and/or 12 varied, while the lipid tail for each was assigned as (6*S*)-methyloctanoyl, as found in TriB_1_ (**86**). No clear structure–activity relationship could be drawn from the range of analogues isolated, except that there may exist a synergistic relationship between certain amino acids, such that substitutions may impede activity.^24^

The antimicrobial activity across the TriM family (**92–98**) is comparable to tridecaptin A and its variants such that they are active against Gram-negative *E. coli*, *A. baumannii* and even colistin-resistant strains of *K. pneumoniae*. TriM_11_ (**98**) displayed the most potent antimicrobial activity and was even found to be highly active (MIC = 4 μg mL^−1^) against the polymyxin-resistant bacteria, *Proteus mirabilis* and *Serratia marcescens*. Unfortunately, this superior activity is deterred by the high levels of haemolysis found for TriM_11_ (**98**) (∼50%), which is the highest reported across the analogues tested.

Membrane interaction and depolarisation assays were also conducted to ascertain the mode of action of this novel tridecaptin family. Uptake of *N*-(1-naphthyl)aniline (NPN) dye by various Gram-negative strains confirmed OM binding by both TriM_1_ and M_11_, while DiSC_3_(5) membrane depolarization assays are said to align with previous findings of disruption of the proton motive force.^24,30^ Although further mechanism of action studies are required to confirm the cellular interactions responsible for the antimicrobial activity of the TriM variants, this novel class of NRAP- further highlights the potential of the tridecaptins as novel therapeutics for the treatment of stubborn bacterial infections.

## 5. Conclusions and future outlooks

The current output of tridecaptin research has included isolation from nature as well as rational design of synthetic analogues in order to tackle the ever-growing threat of antimicrobial resistance. The nature of biosynthesis of tridecaptins favours their development as antimicrobial peptides as the class have a diverse structural capacity and display potent activity against many strains of multidrug-resistant Gram-negative bacteria. Tridecaptin variants studied to date are also reported to exhibit low levels of resistance development when compared to other clinically relevant antibiotics and have an innate biological profile that poses the potential for safe human administration. Moreover, unacetylated variants H-TriA_1_ and B_1_ have been proven to be effective sensitizers of problematic MDR bacteria when co-administered or covalently linked to other clinically relevant antibiotics, improving their efficacy. They have even found broader application in accurate differential staining of Gram-negative bacteria.

Extensive development of novel synthetic tridecaptin analogues has presented cost-effective antimicrobial peptides with resistance to emerging d-peptidase enzymes that are intrinsically straightforward to synthesize. A complex mechanism of action has been identified for the tridecaptin class, in which lipid II has been confirmed as the chiral inner membrane receptor. The importance of this precursor in peptidoglycan biosynthesis suggests the chance for resistance development is low compared to other antibiotic candidates. While bacteria are usually a step ahead in the continued arms race to tackle antimicrobial resistance, continued research and development into the tridecaptin class and their subsequent implementation into the clinical setting could go a long way to overcome the increasing threat of Gram-negative bacterial infections to human health and global economies.

## Funding sources

We thank the Engineering and Physical Sciences Research Council (EPSRC) and the Department for the Economy (DfE) for their respective funding of Samantha Bann's and Ross Ballantine's PhD studentships.

## Conflicts of interest

There is no conflict of interest to declare.

## Supplementary Material

MD-012-D0MD00413H-s001
