# Supplementary material for: The tridecaptins: non-ribosomal peptides that selectively target Gram-negative bacteria
Source: RSC Med Chem. 2021 Jan 22;12(4):538–51. doi: 10.1039/d0md00413h (PMC8127968; doi:10.1039/d0md00413h)
Supplement: MD-012-D0MD00413H-s001 [file MD-012-D0MD00413H-s001.pdf]

Electronic Supplementary Material (ESI) for RSC Medicinal Chemistry.
This journal is © The Royal Society of Chemistry 2021

Supplementary Information

FOR

The Tridecaptins: Non-Ribosomal Peptides That Selectively Target Gram-Negative Bacteria

Samantha J. Bann,[a] Ross D. Ballantine[a] and Stephen A. Cochrane*[a]

[a] School of Chemistry and Chemical Engineering, David Keir Building, Stranmillis Road, Queen's University Belfast, Belfast, UK, BT9 5AG

*To whom correspondence should be addressed. Email: s.cochrane@qub.ac.uk

Contains chemical structures of all natural and synthetic tridecaptin analogues reported to date, with MIC data and also toxicity and haemolytic data, where available.

TABLE OF CONTENTS

**Contents**	**Page Number**
Analogues **1** – **4**:<br>J. Shoji *et al.*, *J. Antibiot.* **1978**, *31* (7), 646–651.<br>T. Kato *et al.*., *J. Antibiot.* **1978**, *31*, 652–661.<br>J. Shoji *et al.*, *J. Antibiot.* **1979**, *32* (4), 313–319.<br>C. T. Lohans *et al.*, *J. Am. Chem. Soc.* **2012**, *134* (48), 19540–19543.	S1, S3
Analogues **5** – **8**:<br>J. Shoji *et al.*, *J. Antibiot.* **1978**, *31* (7), 646–651.<br>T. Kato *et al.*, *J. Antibiot.* **1979**, *32* (4), 305–312.<br>J. Shoji *et al.*, *J. Antibiot.* **1979**, *32* (4), 313–319.	S2, S3
Analogues **9** – **11**:<br>J. Shoji *et al.*, *J. Antibiot.* **1978**, *31* (7), 646–651.<br>T. Kato *et al.*, *J. Antibiot.* **1979**, *32* (4), 305–312.<br>J. Shoji *et al.*, *J. Antibiot.* **1979**, *32* (4), 313–319.	S2, S3
Analogues **1**, **12** – **32**: S. A. Cochrane *et al.*, *J. Med. Chem.* **2014**, 57, 1127–1131.	S4
Analogues **33** – **40**: S. A. Cochrane *et al.*, *J. Med. Chem*. **2015**, 58 (24), 9779–9785.	S9
Analogues **41** – **53**: S. A. Cochrane *et al.*, *ChemBioChem* **2014**, 15 (9), 1295–1299.	S13
Analogues **54** – **61**: R. D. Ballantine *et al.*, *Chem. Commun*. **2018**, 54, 10634–10637.	S16
Analogues **62** – **68**: R. D. Ballantine *et al.*, *MedChemComm* **2019**, 10, 484–487.	S18
Analogues **69** – **84**: S. J. Bann *et al.*, *J. Med. Chem.* **2019**, 62, 10466–10472.	S21
Analogue **85**: W. Wang *et al.*, *Anal. Bioanal. Chem.* **2019**, 411, 4017–4023.	S25
Analogues **86** – **91**: S. A. Cochrane *et al.*, *Org. Biomol. Chem.* **2015**, 13 (21), 6073–6081.	S26
Analogue **92**: M. Jangra *et al.*, *Antimicrob. Agents Chemother.* **2019**, 63 (6), e00338-19.	S28
Analogues **93** – **98**: M Jangra *et al.*, *Sci. Rep.* **2019**, 9 (18870), doi.org/10.1038/s41598-019-54716-8.	S30

Isolation of Tridecaptins A, B and C
J. Shoji, H. Hinoo, R. Sakazaki, T. Kato, Y. Wakisaka, M. Mayama, S. M. and H. M.
J. Antibiot. **1978**, 31 (7), 646-651.

The Structure of Tridecaptin A (Studies on Antibiotics From the Genus Bacillus. XXIV)
Kato, T., Hinoo, H., Shoji, J. *J. Antibiot.* **1978**, 31, 652-661.

The Structures of Tridecaptins B and C
Kato, T.; Sakazaki, R.; Hinoo, H.; Shoji, J. *J. Antibiot.* **1979**, 32 (4), 305-312.

Resolution of Peptide Antibiotics, Cerexins and Tridecaptins, By High Performance Liquid Chromatography (Studies on Antibiotics From the Genus Bacillus. XXVI)
Shoji, J.; Kato, T.; Terabe, S.; Konaka, R. *J. Antibiot.* **1979**, 32 (4), 313-319.

Structural Characterization of the Highly Cyclized Lantibiotic Paenicidin A via a Partial Desulfurization/Reduction Strategy
Lohans, C. T.; Huang, Z.; Van Belkum, M. J.; Giroud, M.; Sit, C. S.; Steels, E. M.; Zheng, J.; Whittal, R. M.; McMullen, L. M.; Vederas, J. C. *J. Am. Chem. Soc.* **2012**, 134 (48), 19540-19543.

Tridecaptin A analogues were isolated as mixtures of fatty acid variants.

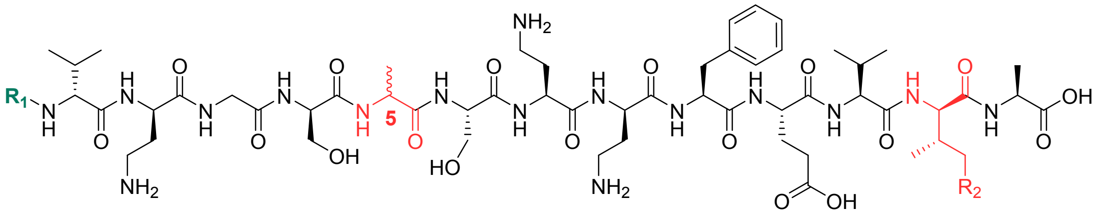

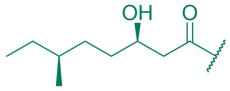

***ai*C$_9$h^3**

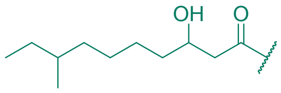

***ai*C$_{11}$h^3**

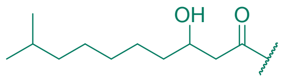

***i*C$_{11}$h^3**

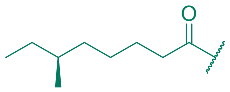

***ai*C$_9$**

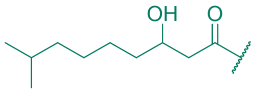

***i*C$_{10}$h^3**

1a. TriA$_{\alpha(1)}$: R$_1$ = *ai*C$_9$h^3, R$_2$ = Me, 5 = D-Trp
1b. TriA$_{\alpha(1)}$: R$_1$ = *ai*C$_{11}$h^3, R$_2$ = Me, 5 = D-Trp
1c. TriA$_{\alpha(1)}$: R$_1$ = *i*C$_{11}$h^3, R$_2$ = Me, 5 = D-Trp

2. TriA$_{\beta(2)}$: R$_1$ = *ai*C$_9$h^3, R$_2$ = H, 5 = D-Trp

3a. TriA$_3$: R$_1$ = *ai*C$_{11}$h^3, R$_2$ = Me, 5 = D-Trp
3b. TriA$_3$: R$_1$ = *i*C$_{11}$h^3, R$_2$ = Me, 5 = D-Trp

4a. TriA$_4$: R$_1$ = *ai*C$_{11}$h^3, R$_2$ = Me, 5 = D-Phe
4b. TriA$_4$: R$_1$ = *i*C$_{11}$h^3, R$_2$ = Me, 5 = D-Phe

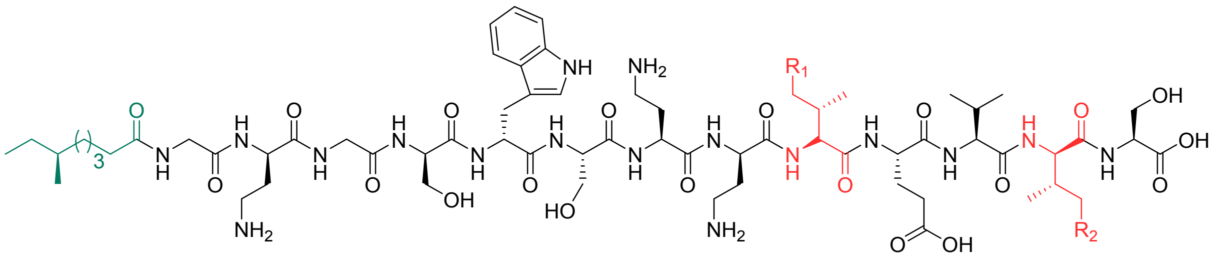

5. TriB$_{\alpha}$ R_1: Me, R_2: Me

6. TriB$_{\beta}$ R_1: Me, R_2: H

7. TriB$_{\gamma}$ R_1: H, R_2: Me

8. TriB$_{\delta}$ R_1: H, R_2: H

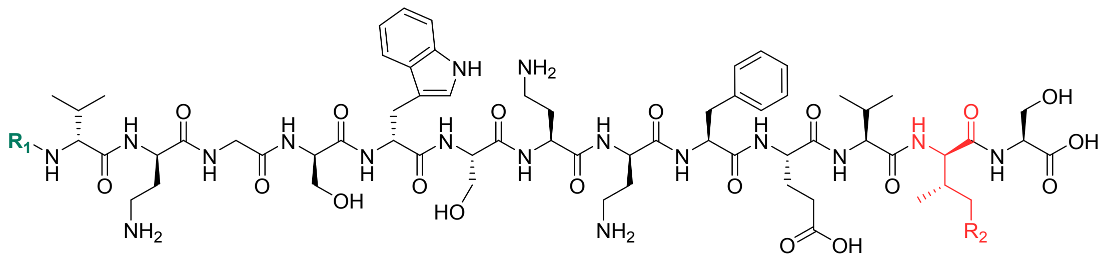

9. TriC$_{\alpha 1}$: R_1 = *ai*$C_{11}h^3$, R_2 = H **10.** TriC$_{\alpha 2}$: R_1 = *i*$C_{10}h^3$, R_2 = H **11.** TriC$_{\beta 1}$: R_1 = *ai*$C_{11}h^3$, R_2 = Me

Table S1. MIC values of TriA, B and C analogues against various bacterial strains.

J. Shoji, H. Hinoo, R. Sakazaki, T. Kato, Y. Wakisaka, M. Mayama, S. M. and H. M. J. Antibiot. ***1978,*** *31 (7), 646–651.*			
	**Analogue**		
Bacterial strain and associated MIC (μg/mL)	A	B	C
Bacillus subtilis **PCI 219**	12.5	12.5	6.25
Staphylococcus aureus **FDA 209P JC-1**	50	25	12.5
S. aureus **Smith**	50	25	12.5
S. aureus **80257**	50	25	12.5
Streptococcus pyogenes **C-203**	50	25	12.5
Diplococcus pneumoniae	>50	50	25
Escherichia coli **NIHJ JC-2**	6.25	12.5	6.25
E. coli **EC-14**	3.13	6.25	6.25
E. coli **80750**	6.25	6.25	3.13
Klebsiella pneumoniae	6.25	12.5	6.25
Salmonella typhimurium	6.25	12.5	6.25
Pseudomonas aeruginosa Ps**-24**	50	50	25
Proteus vulgaris **CN-329**	50	>50	>50
Proteus mirabilis **PR-4**	>50	>50	>50

Synthesis and Structure-Activity Relationship Studies of N-Terminal Analogues of the Antimicrobial Peptide Tridecaptin A_1

Cochrane, S. A.; Lohans, C. T.; Brandelli, J. R.; Mulvey, G.; Armstrong, G. D.; Vederas, J. C. *J. Med. Chem.* **2014**, 57, 1127-1131.

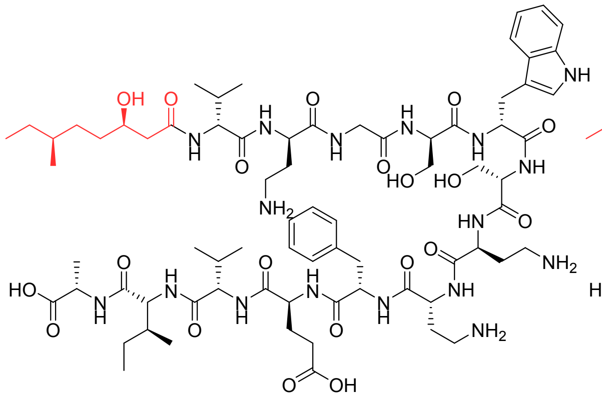

1. Natural $TriA_1$

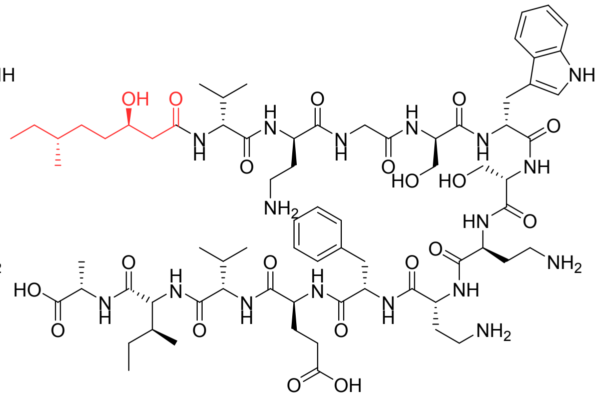

12. (3*R*,6*R*)-$TriA_1$

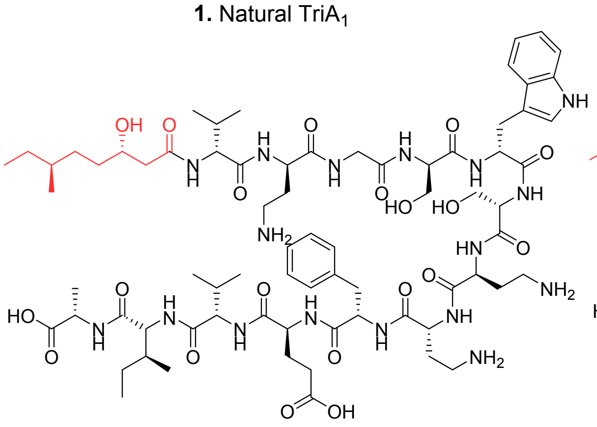

13. (3*S*,6*S*)-$TriA_1$

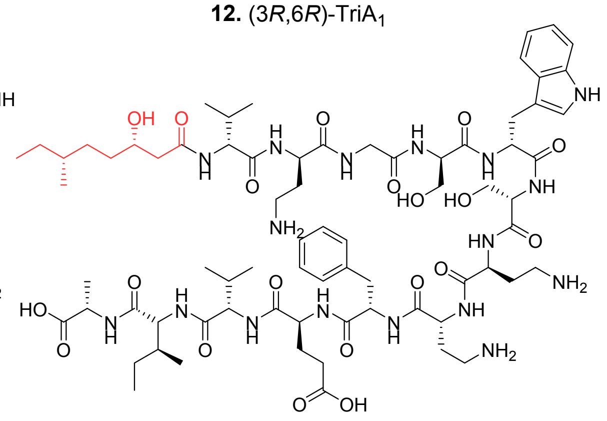

14. (3*S*,6*R*)-$TriA_1$

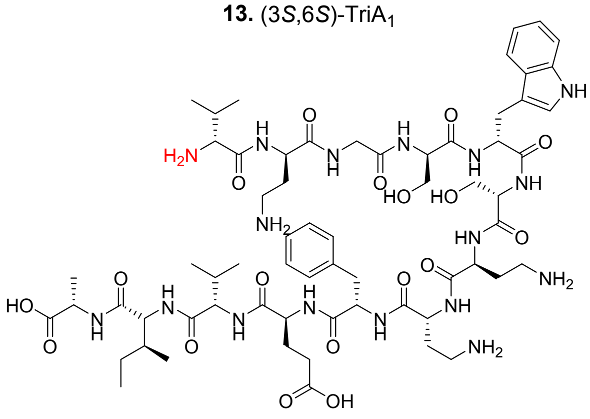

15. H-$TriA_1$

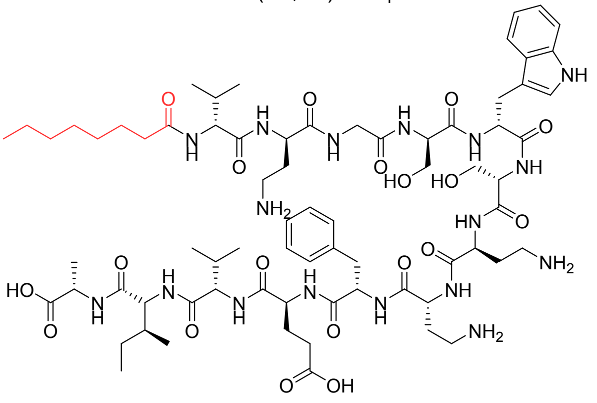

16. Oct-$TriA_1$

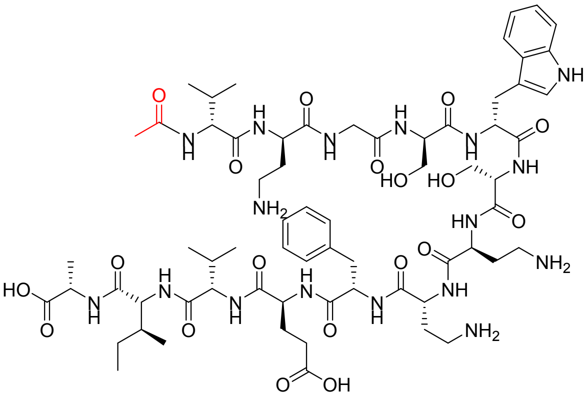

17. C2-$TriA_1$

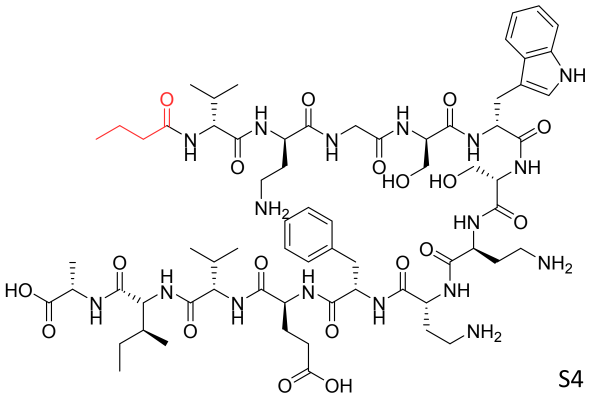

18. C4-$TriA_1$

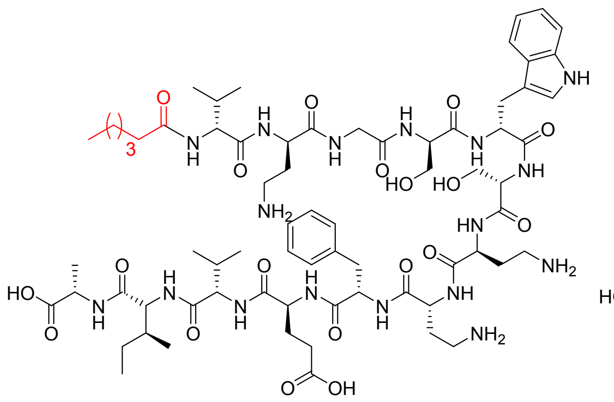

19. C6-TriA$_1$

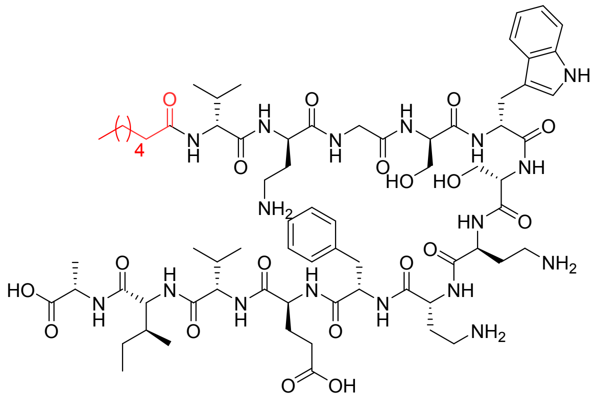

20. C7-TriA$_1$

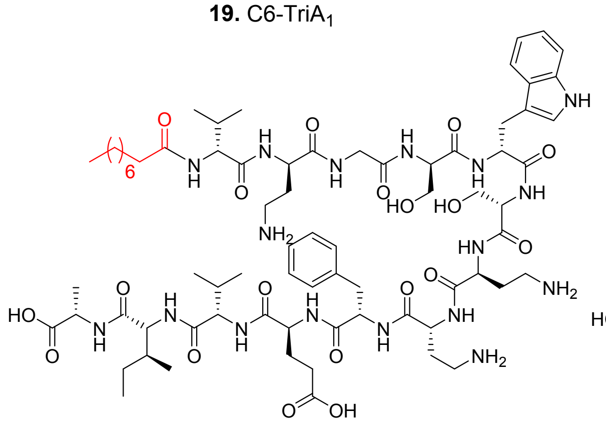

21. C9-TriA$_1$

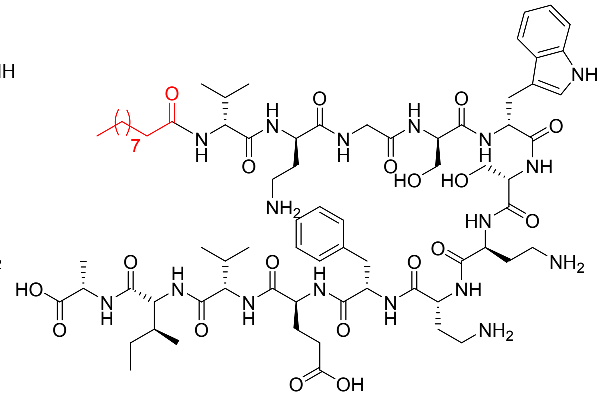

22. C10-TriA$_1$

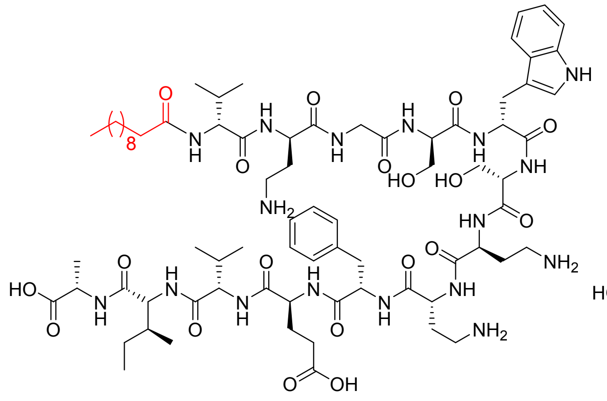

23. C11-TriA$_1$

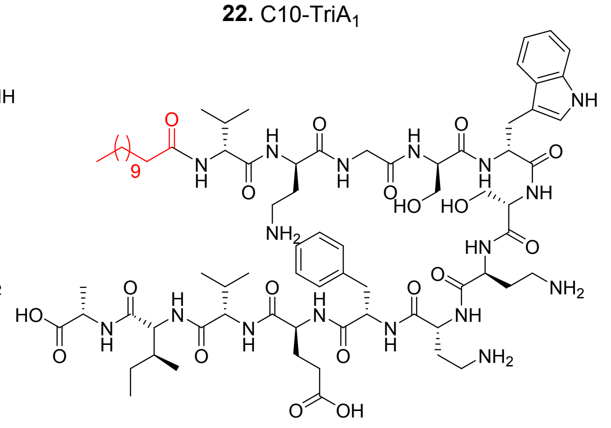

24. C12-TriA$_1$

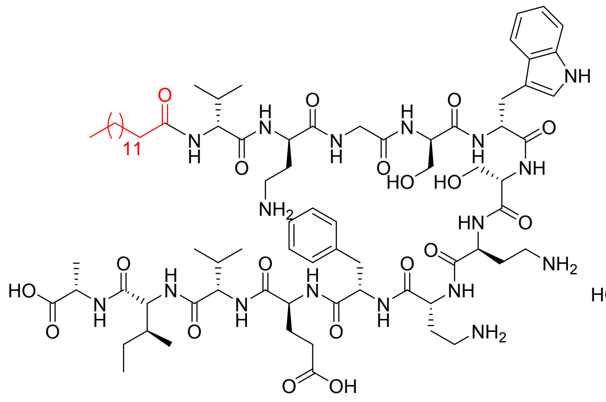

25. C14-TriA$_1$

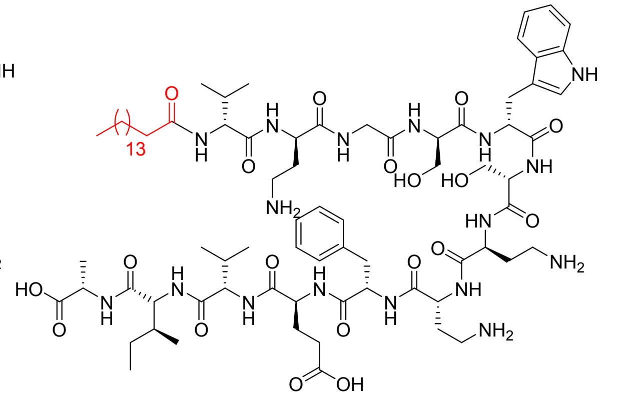

26. C16-TriA$_1$

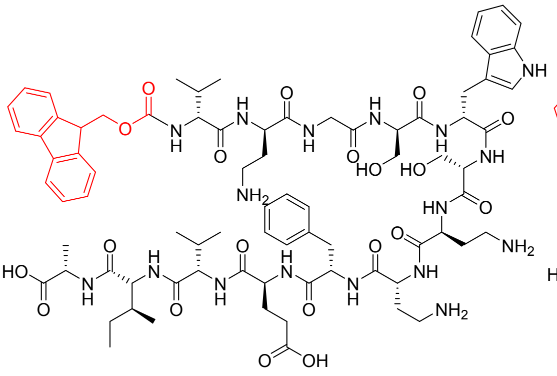

27. Fmoc-TriA$_1$

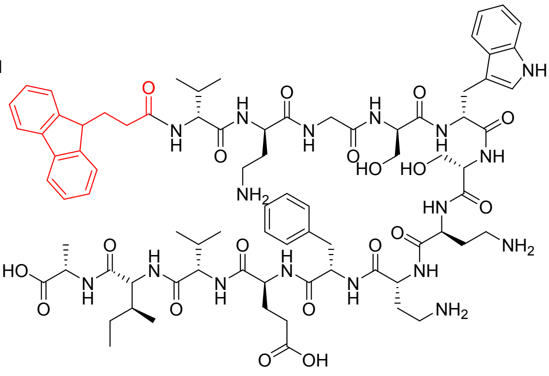

28. Fpa-TriA$_1$

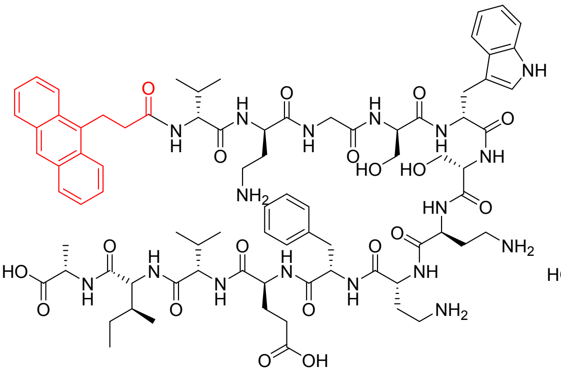

29. Apa-TriA$_1$

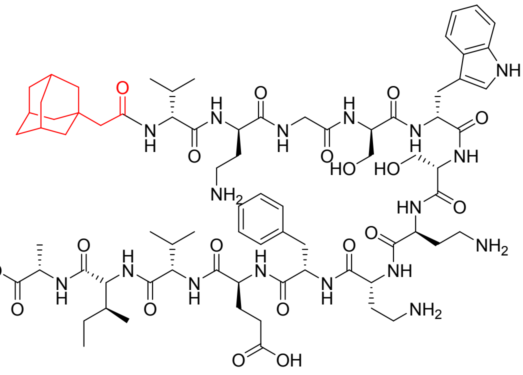

30. Aaa-TriA$_1$

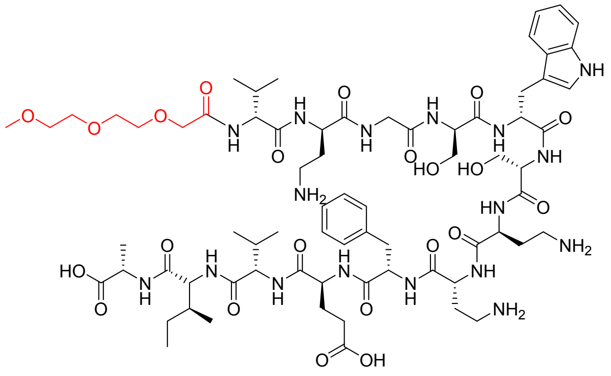

31. PEG-TriA$_1$

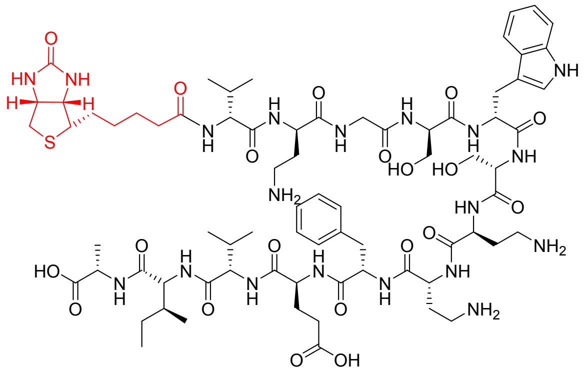

32. Bio-TriA$_1$

Cochrane, S. A.; Lohans, C. T.; Brandelli, J. R.; Mulvey, G.; Armstrong, G. D.; Vederas, J. C. J. Med. Chem. ***2014****, 57, 1127–1131.*										
**Analogue**	**MIC (μg/mL)**									
	Escherichia coli ATCC 25922	*Salmonella enterica ATCC 13311*	*Pseudomonas aeruginosa* ATCC 27853	*Campylobacter jejuni* NCTC 11168	*Klebsiella pneumoniae* ATCC 13883	*Acinetobacter baumannii* ATCC 19606	*Enterococcus faecalis* ATCC 29212	*Staphylococcus aureus* ATCC 29213	*Listeria monocytogenes* ATCC 19434	*Enterococcus faecium* ATCC 19434
**1**	3.13	6.25	50	1.56	3.13	12.5	>100	>100	50	>100
**12**	3.13	6.25	50	3.13	6.25	12.5	>100	>100	100	>100
**13**	6.25	6.25	50	1.56	6.25	25	>100	>100	100	>100
**14**	12.5	25	100	3.13	12.5	50	>100	>100	>100	>100
**15**	100	100	100	>100	50	>100	>100	>100	>100	>100
**16**	3.13	6.25	25	0.78	3.13	12.5	100	100	25	50
**17**	50	100	>100	100	50	>100	>100	>100	>100	>100
**18**	12.5	25	>100	12.5	25	100	>100	>100	>100	>100
**19**	3.13	6.25	50	3.13	3.13	25	>100	>100	100	>100
**20**	6.25	12.5	50	1.56	6.25	12.5	>100	>100	50	>100
**21**	12.5	6.25	100	0.78	12.5	50	100	100	25	100
**22**	6.25	12.5	>100	0.4	6.25	12.5	>100	>100	25	100
**23**	6.25	12.5	>100	0.4	6.25	12.5	>100	>100	25	100
**24**	12.5	50	>100	0.4	12.5	50	>100	>100	50	100
**25**	>100	>100	>100	12.5	>100	>100	>100	>100	>100	>100
**26**	>100	>100	>100	50	>100	>100	>100	>100	>100	>100
**27**	6.25	6.25	>100	0.78	6.25	12.5	50	100	12.5	25
**28**	6.25	>100	>100	1.56	100	>100	>100	>100	100	>100
**29**	6.25	12.5	>100	1.56	12.5	25	>100	>100	50	>100
**30**	6.25	6.25	50	1.56	12.5	12.5	50	50	25	50
**31**	>100	>100	>100	>100	>100	>100	>100	>100	>100	>100
**32**	50	50	>100	50	50	>100	>100	>100	>100	>100

Table S2. MIC values of N-Terminal $TriA_1$ analogues against various bacterial strains.

Table S3. Toxicity data for N-Terminal TriA$_1$ analogues.

Cochrane, S. A.; Lohans, C. T.; Brandelli, J. R.; Mulvey, G.; Armstrong, G. D.; Vederas, J. C. J. Med. Chem. ***2014****, 57, 1127–1131.*		
**Analogue**[a]	**% haemolysis**[b]	**IC_{50} (μg/mL)**[c]
**1**	3.2	200
**15**	0.5	
**16**	4.7	100
**18**	0.8	
**24**	82.2	
**27**	100	

[a] All peptides were tested at 83 μg/mL. [b] Haemolytic assays were run in triplicate. Absorbance of each sample was measured at 415 nm and percent haemolysis due to the corresponding peptide was calculated relative to Triton X-100 (taken as 100%). [c] Cytotoxicity was determined against HEK 293 cell line with the respective absorbance of each cell measured at 570 nm.

Synthesis of Tridecaptin-Antibiotic Conjugates with in Vivo Activity against Gram-Negative Bacteria
Cochrane, S. A.; Li, X.; He, S.; Yu, M.; Wu, M.; Vederas, J. C. *J. Med. Chem.* **2015**, 58 (24), 9779-9785.

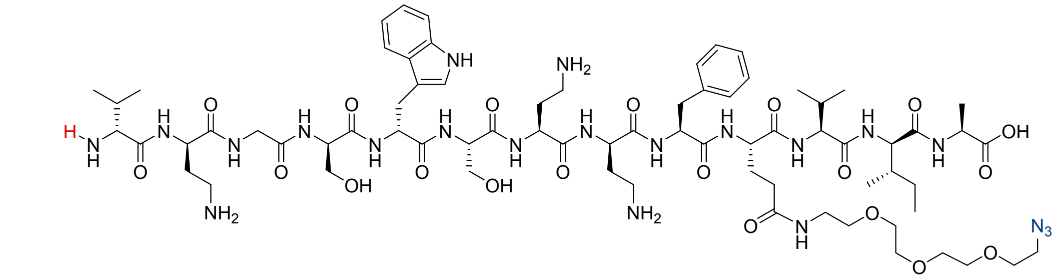

33. H-TriA$_1$(Glu10-PEG$_3$N$_3$)

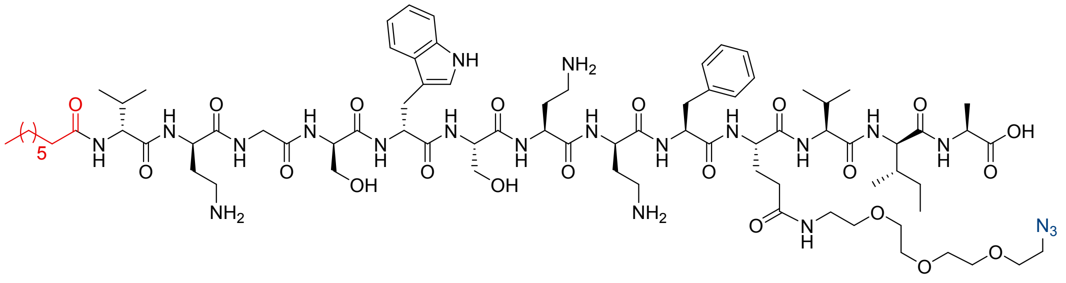

34. Oct-TriA$_1$(Glu10-PEG$_3$N$_3$)

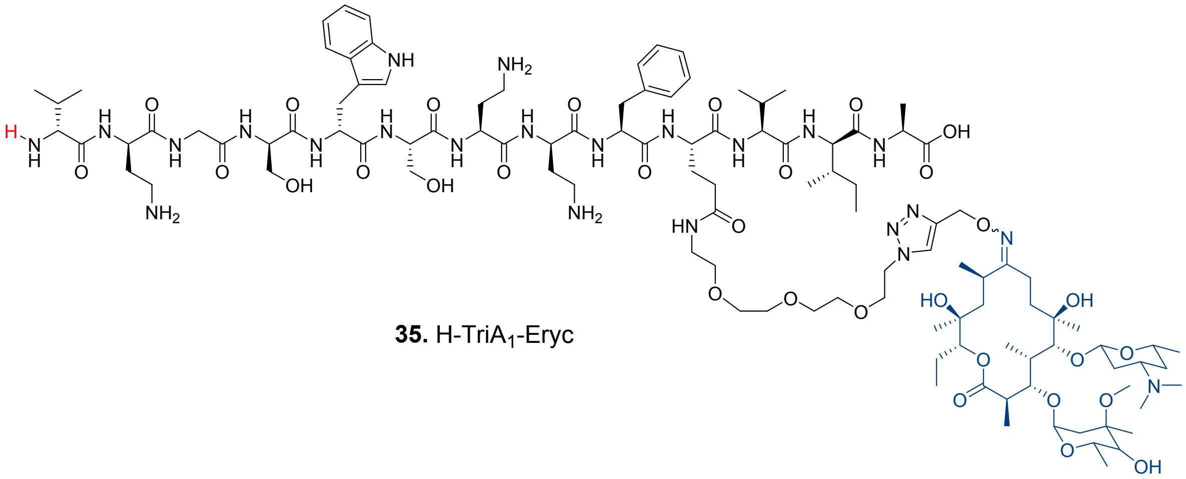

35. H-TriA$_1$-Eryc

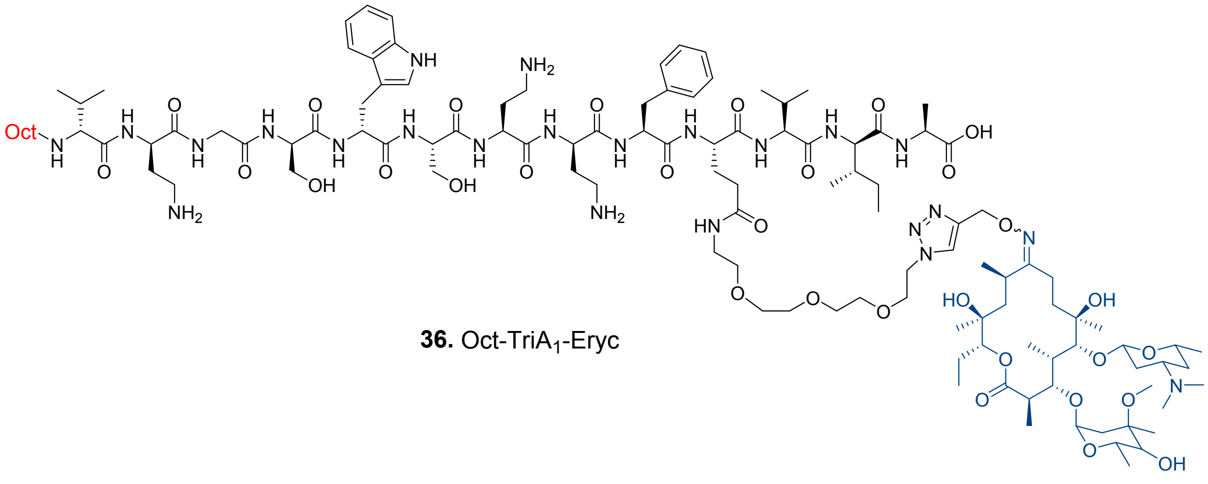

36. Oct-TriA$_1$-Eryc

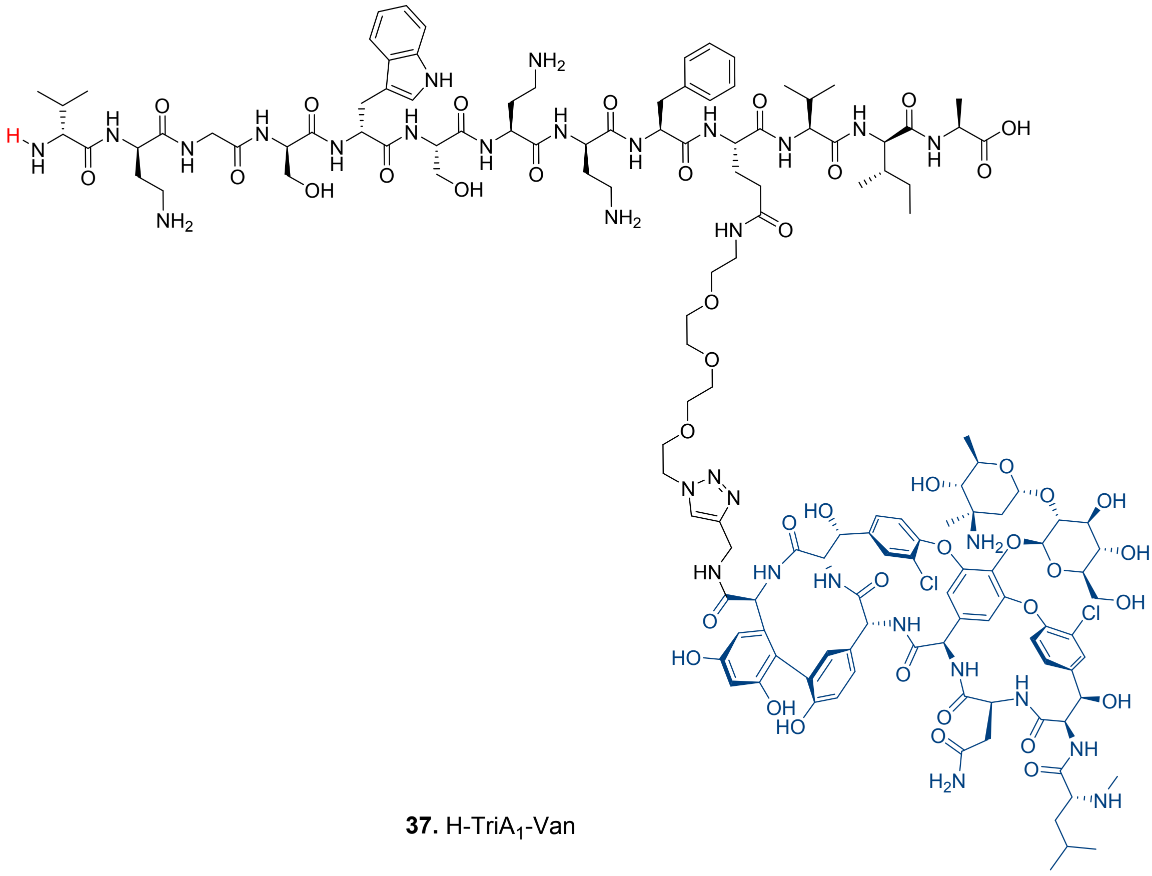

37. H-TriA$_1$-Van

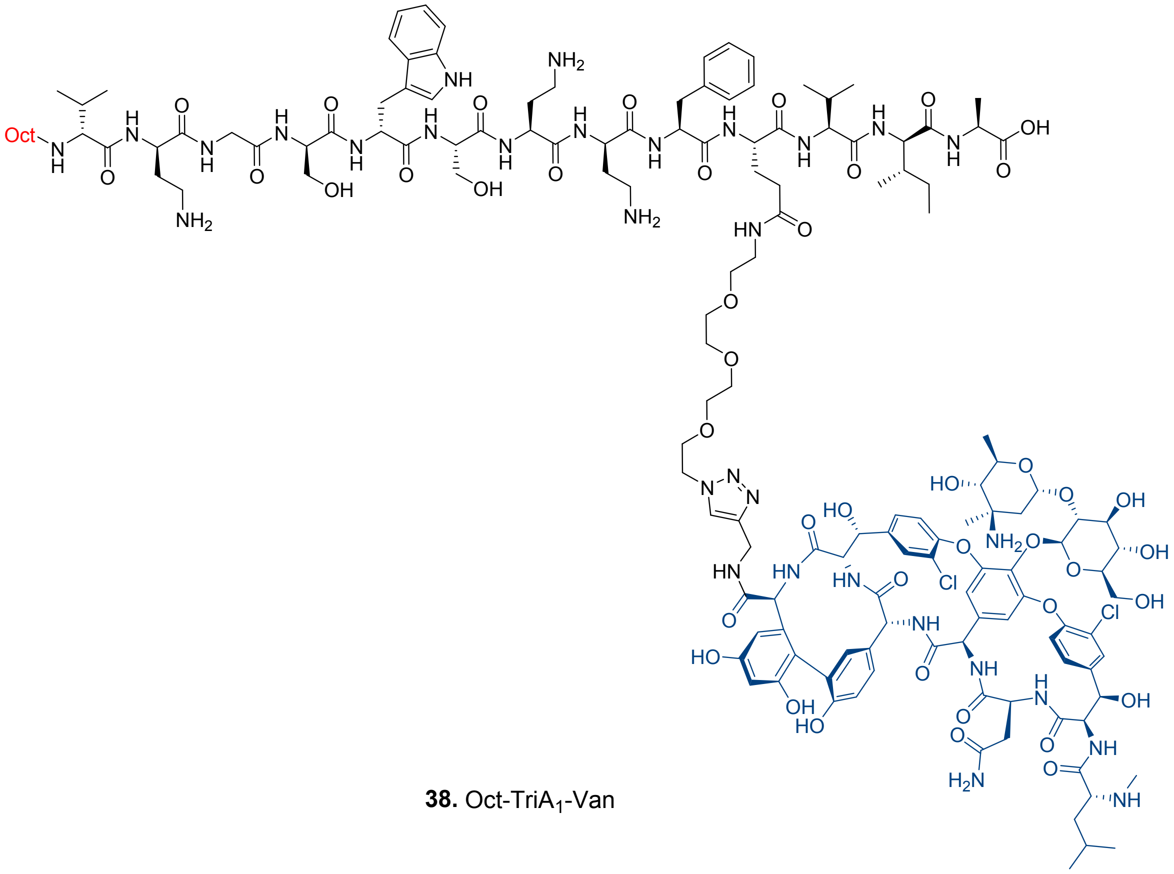

38. Oct-TriA$_1$-Van

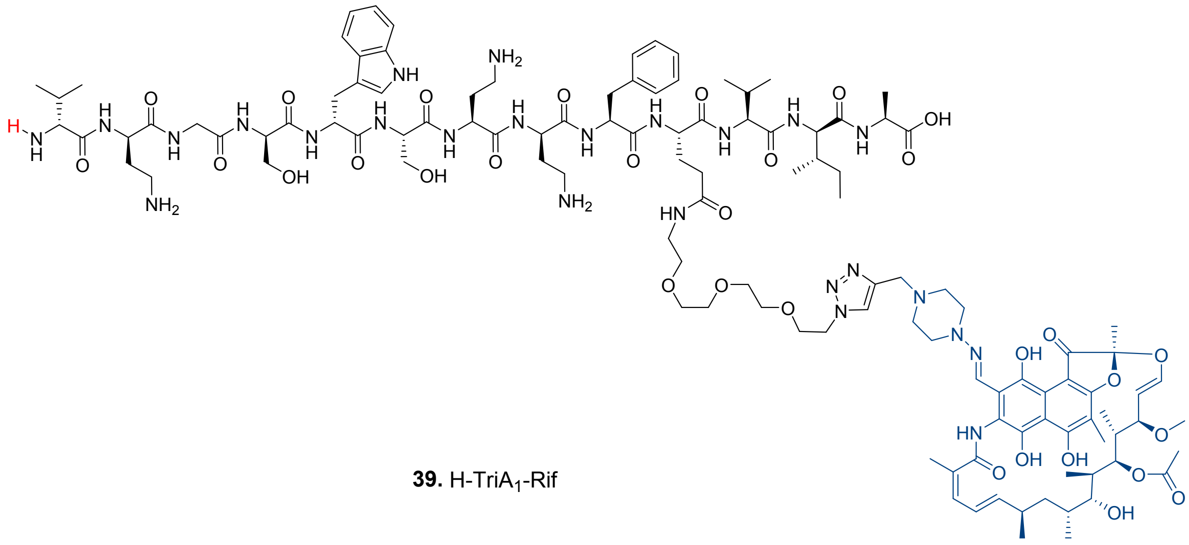

39. H-TriA$_1$-Rif

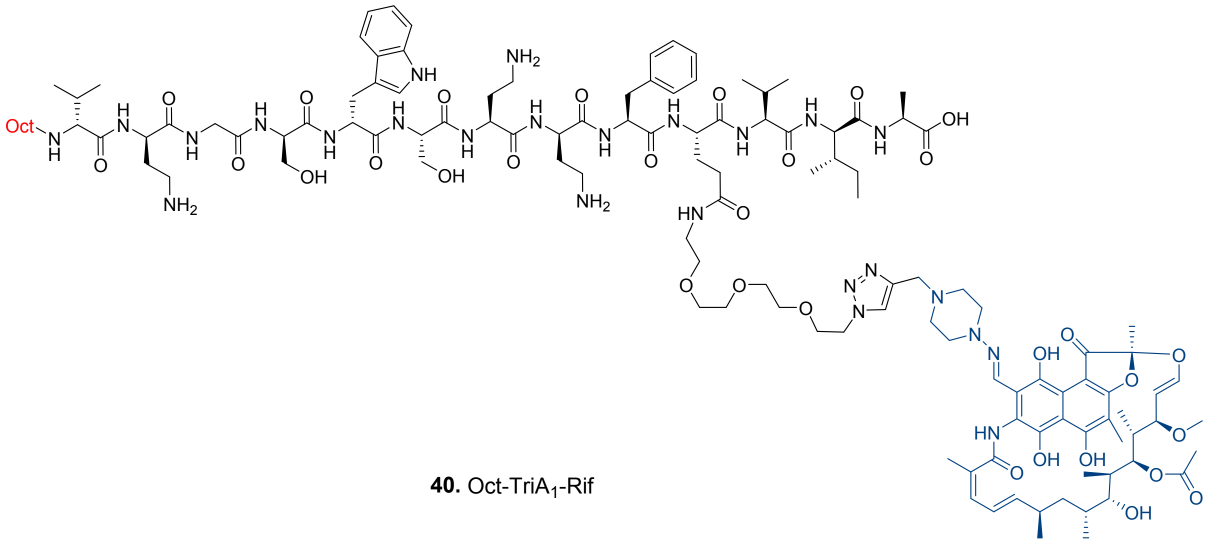

40. Oct-TriA$_1$-Rif

Table S4. MIC values of Tridecaptin-Antibiotic conjugates against various bacterial strains.

Cochrane, S. A.; Li, X.; He, S.; Yu, M.; Wu, M.; Vederas, J. C. J. Med. Chem. ***2015****, 58 (24), 9779–9785.*					
	**MIC (μg/mL)**				
**Analogue**	*E. coli* ATCC 25922	*K. pneumoniae* ATCC 13883	MDR *Kp* ATCC 700603	*A. baumannii* ATCC 19606	MDR *Ab* ATCC BAA-1605
**35**	50	50	200	50	25
**36**	12.5	3.13	12.5	6.25	6.25
**37**	25	100	200	25	25
**38**	12.5	100	25	25	50
**39**	25	50	100	25	50
**40**	3.13	6.25	100	6.25	6.25

Key Residues in Octyl-Tridecaptin A_1 Analogues Linked to Stable Secondary Structures in the Membrane

Cochrane, S. A.; Findlay, B.; Vederas, J. C.; Ratemi, E. S. *ChemBioChem* **2014**, 15 (9), 1295-1299.

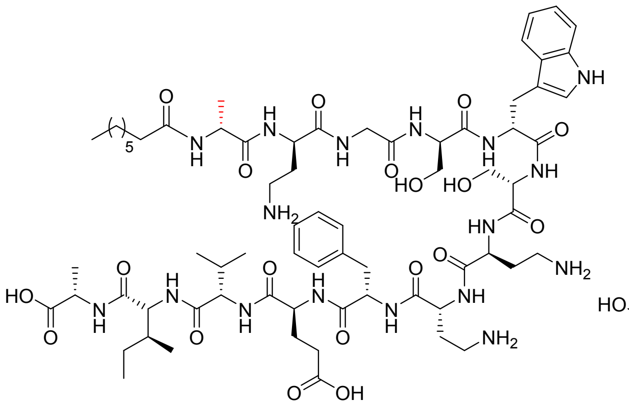

41. Oct-TriA$_1$ (D-Ala1)

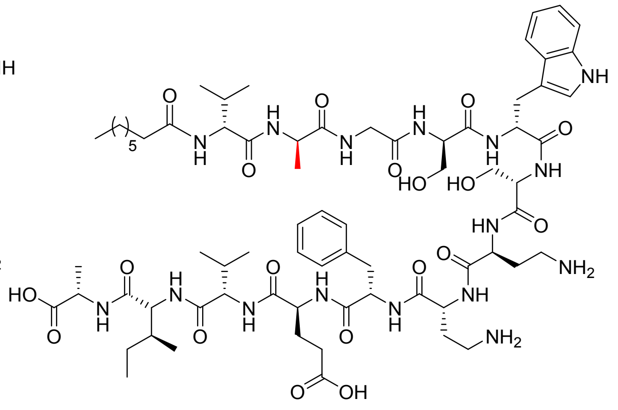

42. Oct-TriA$_1$ (D-Ala2)

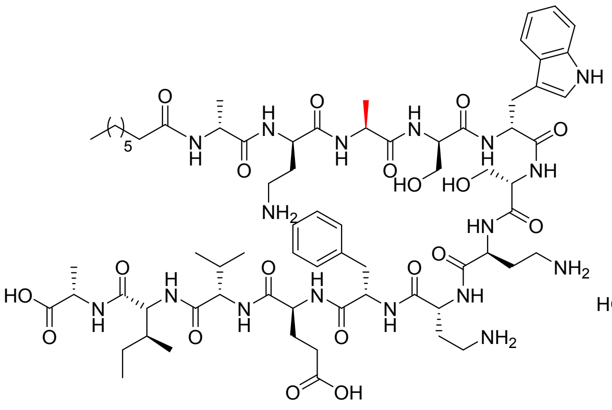

43. Oct-TriA$_1$ (Ala3)

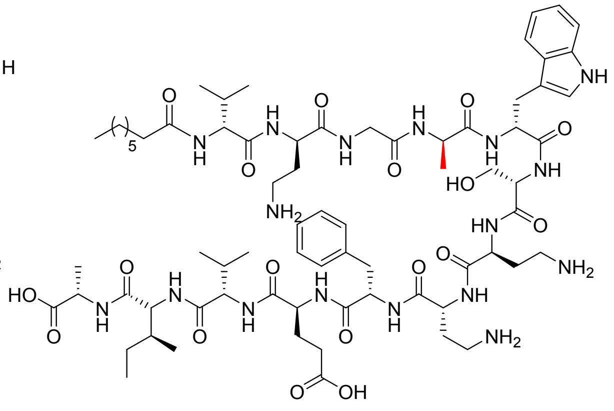

44. Oct-TriA$_1$ (D-Ala4)

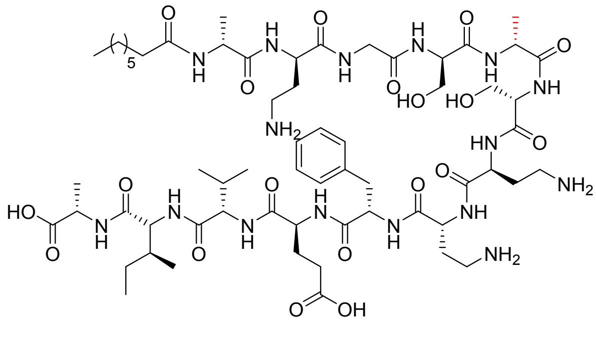

45. Oct-TriA$_1$ (D-Ala5)

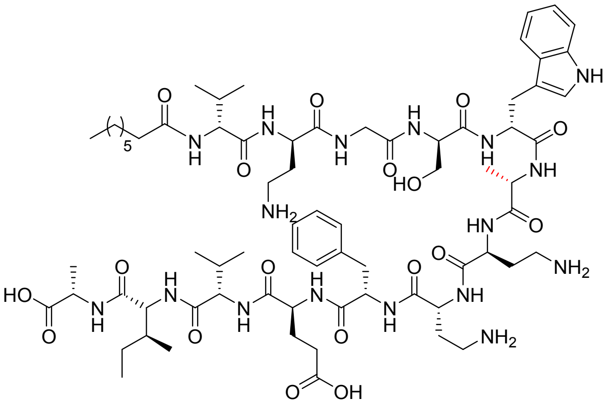

46. Oct-TriA$_1$ (Ala6)

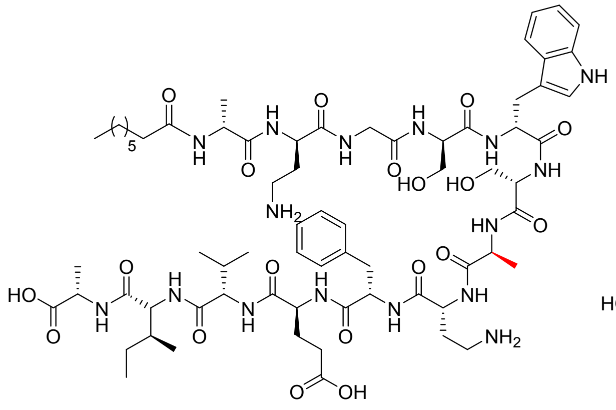

47. Oct-TriA$_1$ (Ala7)

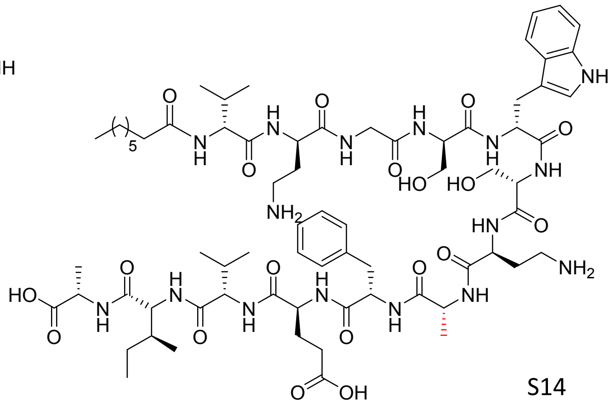

48. Oct-TriA$_1$ (D-Ala8)

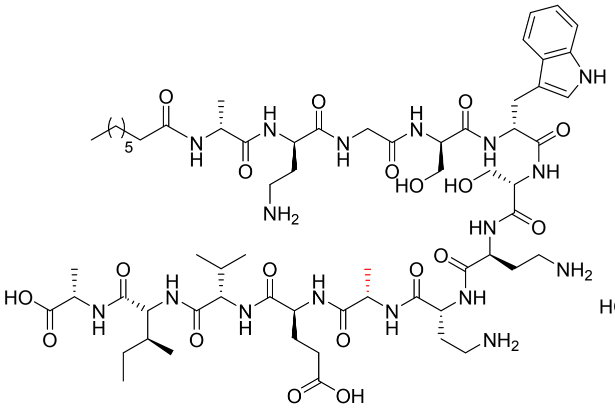

49. Oct-TriA$_1$ (Ala9)

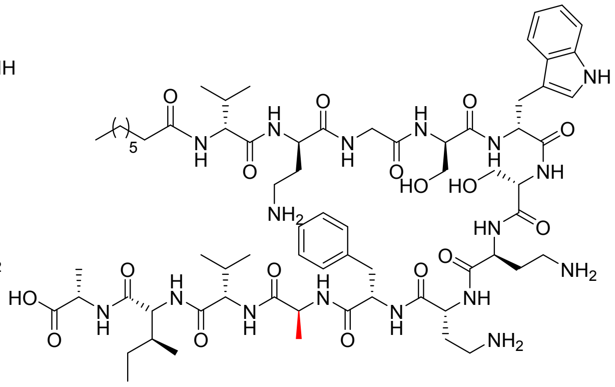

50. Oct-TriA$_1$ (Ala10)

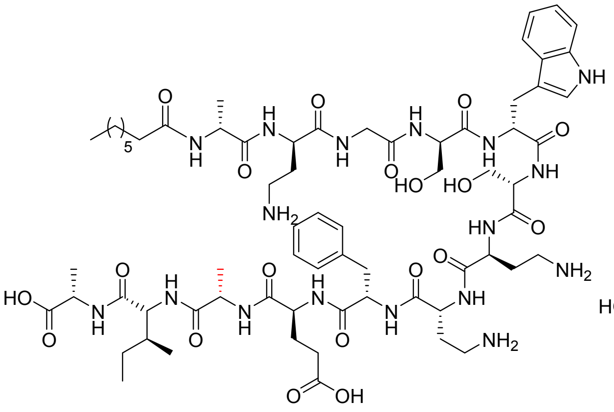

51. Oct-TriA$_1$ (Ala11)

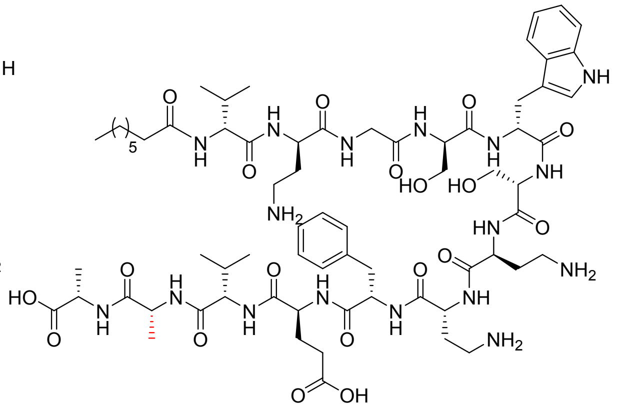

52. Oct-TriA$_1$ (D-Ala12)

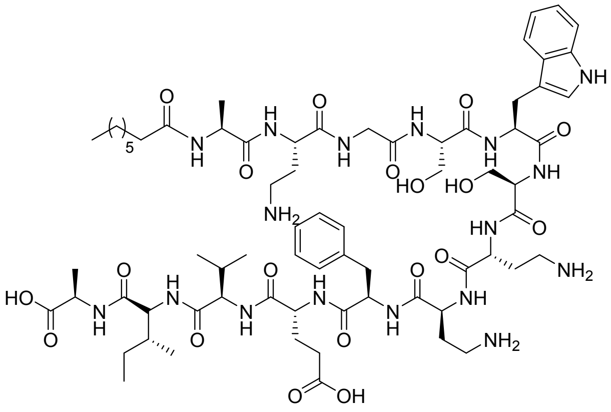

53. *Ent*-Oct-TriA$_1$

Table S5. MIC values of alanine derivatives of Oct-TriA$_1$ against various bacterial strains.

Cochrane, S. A.; Findlay, B.; Vederas, J. C.; Ratemi, E. S. ChemBioChem ***2014****, 15 (9), 1295–1299.*										
	**MIC (μg/mL)**									
**Analogue**	*E. coli* ATCC 25922	*S. enterica* ATCC 13311	*P. aeruginosa* ATCC 27853	*C. jejuni* NCTC 11168	*K. pneumoniae* ATCC 13883	*A. baumannii* ATCC 19606	*E. faecalis* ATCC 29212	*S. aureus* ATCC 29213	*L. monocytogenes* ATCC 15313	*E. faecium* ATCC 19434
**16**	3.13	6.25	25	1.56	3.13	12.5	100	100	25	50
**41**	6.25	6.25	25	1.56	3.13	25	>100	>100	>100	>100
**42**	12.5	12.5	>100	12.5	6.25	>100	>100	>100	>100	>100
**43**	3.13	12.5	100	6.25	6.25	50	>100	>100	>100	>100
**44**	6.25	6.25	25	6.25	6.25	12.5	>100	>100	>100	>100
**45**	25	100	100	50	50	100	>100	>100	>100	>100
**46**	3.13	6.25	100	6.25	6.25	12.5	>100	>100	>100	>100
**47**	12.5	25	>100	12.5	12.5	25	>100	>100	>100	>100
**48**	>100	>100	>100	>100	>100	>100	>100	>100	>100	>100
**49**	12.5	12.5	50	6.25	12.5	100	>100	>100	>100	>100
**50**	6.25	6.25	12.5	3.13	6.25	25	>100	>100	25	>100
**51**	6.25	12.5	25	3.13	6.25	50	>100	>100	>100	>100
**52**	50	25	50	12.5	25	>100	>100	>100	>100	>100
**53**	12.5	12.5	25	12.5	12.5	12.5	100	100	100	100

Rational design of new cyclic analogues of the antimicrobial lipopeptide tridecaptin A_1

Ballantine, R. D.; Li, Y. X.; Qian, P. Y.; Cochrane, S. A. *Chem. Commun.* **2018**, 54, 10634-10637.

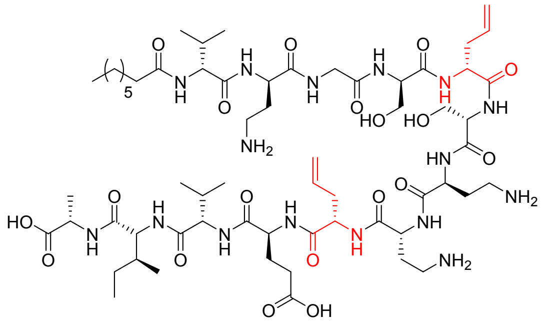

54. Oct-TriA_1 (D-Agl5, Agl9)

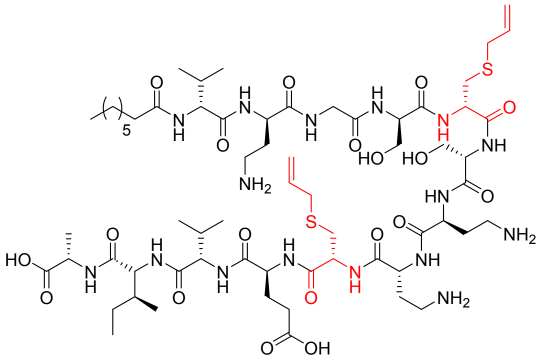

55. Oct-TriA_1 (D-Sac5, Sac9)

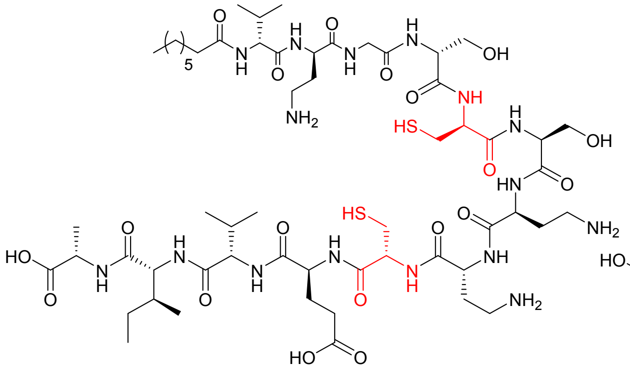

56. Oct-TriA_1 (D-Cys5, Cys9)

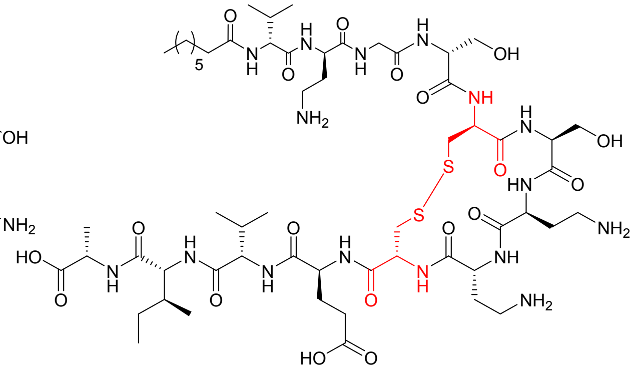

57. Oct-*c*TriA_1 (SS)

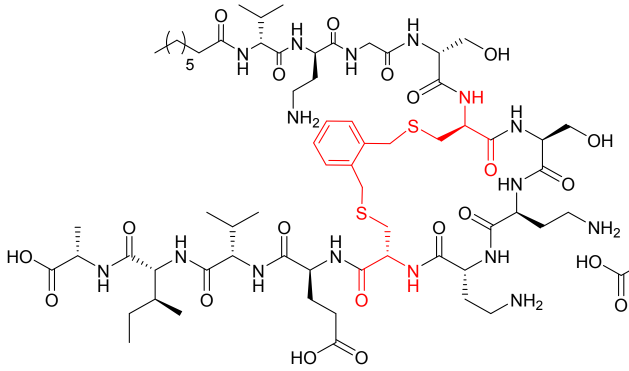

58. Oct-*c*TriA_1 (*o*-Xyl)

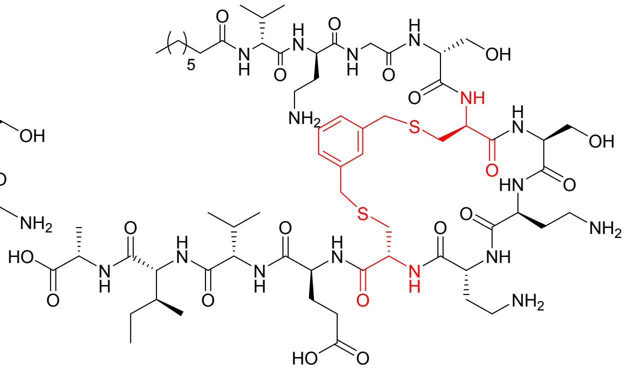

59. Oct-*c*TriA_1 (*m*-Xyl)

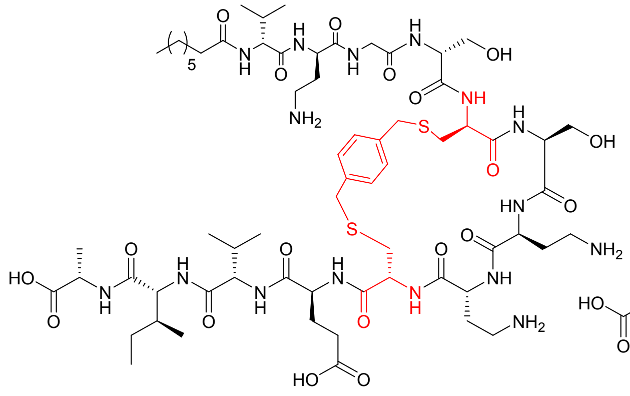

60. Oct-*c*TriA_1 (*p*-Xyl)

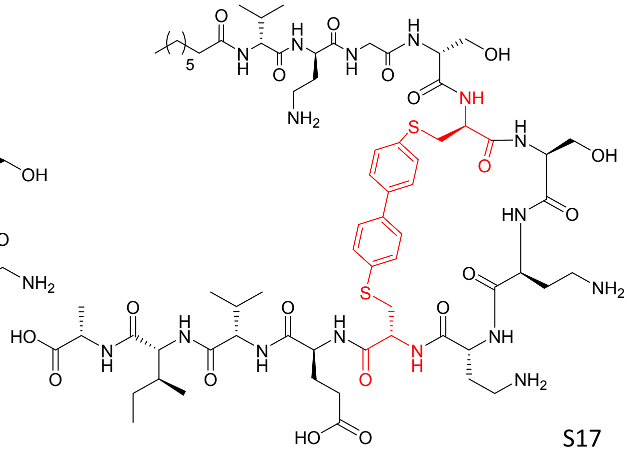

61. Oct-*c*TriA_1 (Biphenyl)

Ballantine, R. D.; Li, Y. X.; Qian, P. Y.; Cochrane, S. A. Chem. Commun. ***2018****, 54, 10634–10637.*					
	**MIC (μg/mL)**				
**Analogue**	*E. coli* NCTC 12241	*S. aureus* NCTC 10788	*K. pneumoniae* NCTC 9633	*A. baumannii* NCTC 13304	*E. cloacae* NCTC 5920
**16**	0.39	25			
**54**	>50	>50			
**55**	>50	>50			
**56**	>50	>50			
**57**	>50	>50			
**58**	6.3	>50	6.3	6.3	12.5
**59**	6.3	>50	6.3	12.5	12.5
**60**	6.3	>50	6.3	12.5	12.5
**61**	>50	>50	>50	>50	>50

Table S6. MIC values of cyclic Oct-TriA$_1$ analogues against various bacterial strains.

Tridecaptin-inspired antimicrobial peptides with activity against multidrug-resistant Gram-negative bacteria

Ballantine, R. D.; McCallion, C. E.; Nassour, E.; Tokajian, S.; Cochrane, S. A.

MedChemComm **2019**, 10, 484-487.

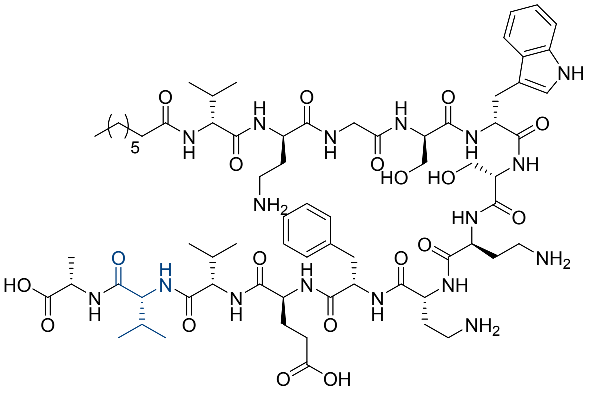

62. Oct-TriA$_2$

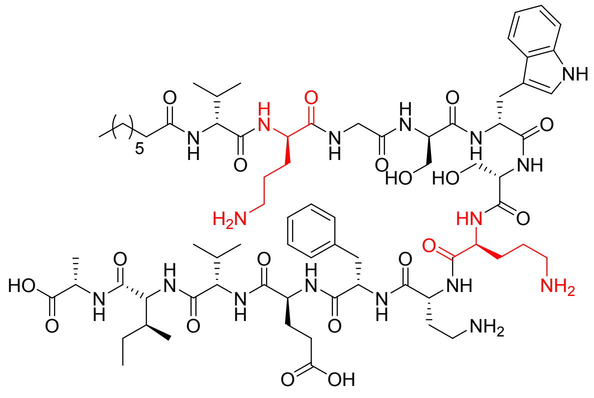

63. Oct-TriA$_1$ (D-Orn2, Orn7)

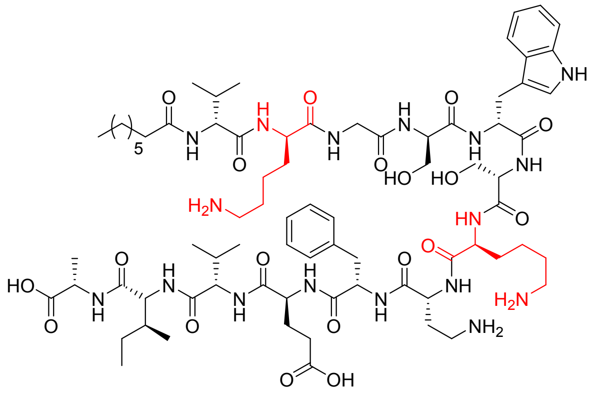

64. Oct-TriA$_1$ (D-Lys2, Lys7)

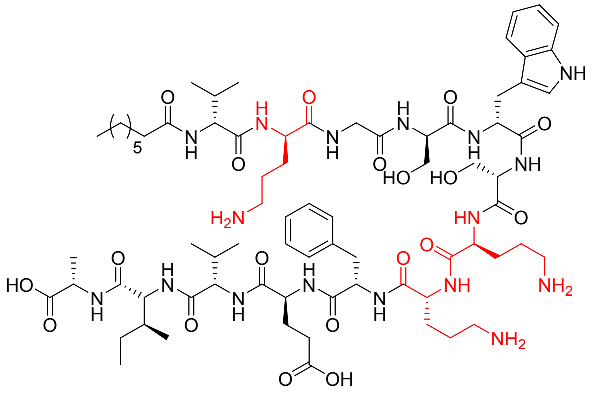

65. Oct-TriA$_1$ (D-Orn2,8, Orn7)

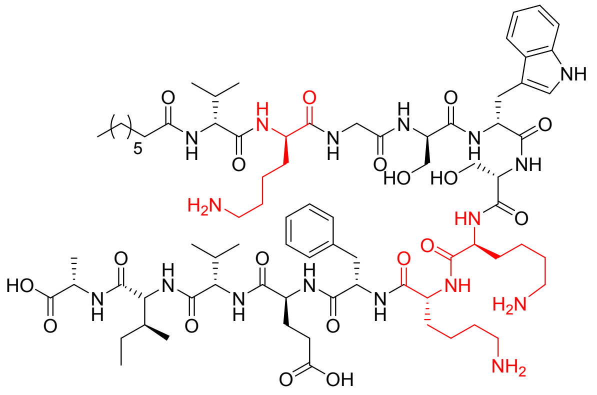

66. Oct-TriA$_1$ (D-Lys2,8, Lys7)

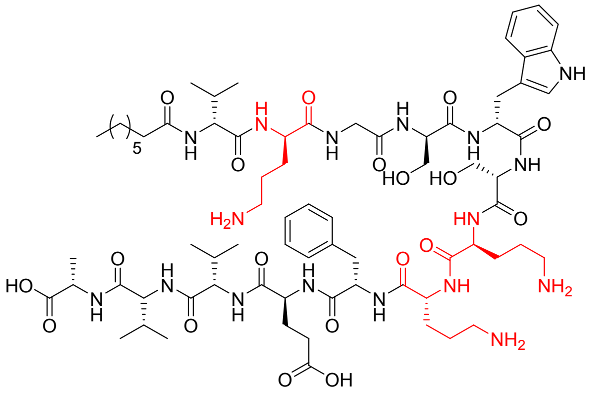

67. Oct-TriA$_2$ (D-Orn2,8, Orn7)

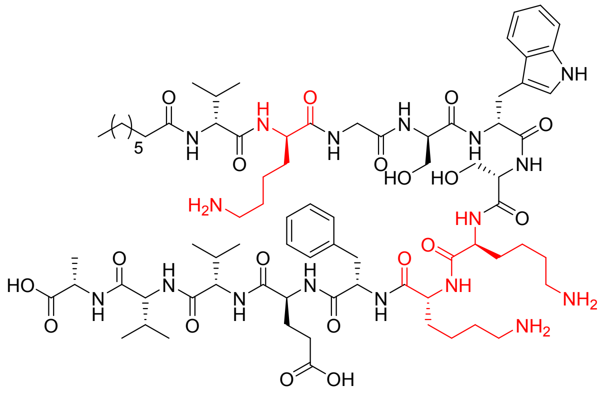

68. Oct-TriA$_2$ (D-Lys2,8, Lys7)

Table S7. MIC values for economical Oct-TriA_1 and A_2 analogues against various bacterial strains.

Ballantine, R. D.; McCallion, C. E.; Nassour, E.; Tokajian, S.; Cochrane, S. A. MedChemComm ***2019****, 10, 484–487.*											
**Analogue**	**MIC (µg/mL)**										
	A. baumannii	*Ab* ACM 11	*Ab* ACM 29	*E. cloacae*	*K. pneumoniae*	*Kp* IMP 170	*Kp* IMP 177	*Kp* IMP 204	*Kp* IMP 216	*Kp* IMP 485	*P. Pseudoalcaligenes*
**16**	12.5	25	25	3.13	6.25	6.25	6.25	12.5	6.25	6.25	50
**62**	25	100	50	6.25	12.5	12.5	25	12.5	12.5	12.5	50
**63**	100	50	50	25	100	25	25	25	25	25	12.5
**64**	50	25	50	25	50	25	25	25	25	25	25
**65**	12.5	50	50	6.25	12.5	25	25	25	6.25	25	50
**66**	6.25	25	25	25	50	50	25	50	25	25	100
**67**	25	25	50	12.5	50	25	12.5	12.5	12.5	12.5	6.25
**68**	>100	>100	>100	>100	>100	>100	>100	>100	>100	>100	>100

Table S8. Haemolytic activity of economical Oct-TriA$_1$ and A$_2$ analogues.

*Ballantine, R. D.; McCallion, C. E.; Nassour, E.; Tokajian, S.; Cochrane, S. A. MedChemComm **2019**, 10, 484–487.*	
**Analogue**[a]	**% haemolysis**[b]
**16**	89.6
**62**	52.1
**63**	66.7
**64**	71.4
**65**	77.5
**66**	86.4
**67**	39.8
**68**	2.8

[a] All peptides were tested at 100 µg/mL. [b] Haemolytic assays were run in triplicate. Absorbance of each sample was measured at 415 nm and percent haemolysis due to the corresponding peptide was calculated relative to Triton X-100 (taken as 100%).

A Chemical-Intervention Strategy To Circumvent Peptide Hydrolysis by D-Stereoselective Peptidases

Bann, S. J.; Ballantine, R. D.; McCallion, C. E.; Qian, P.-Y.; Li, Y.-X.; Cochrane, S. A. *J. Med. Chem.* **2019**, 62, 10466-10472.

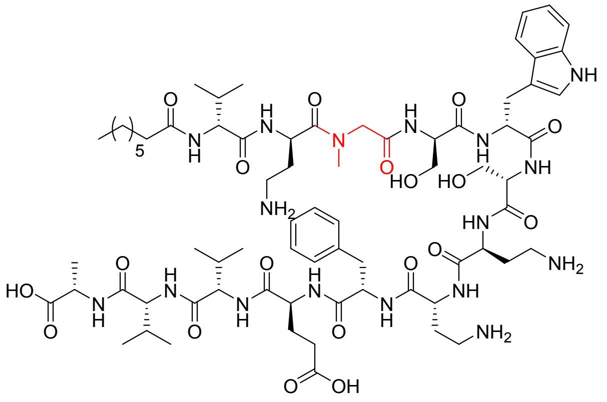

69. Oct-TriA$_2$ (Sar3)

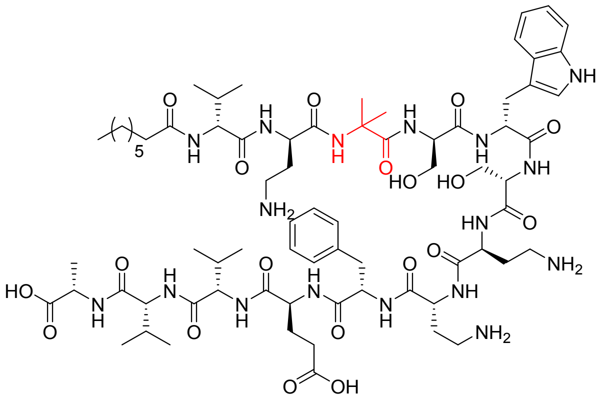

70. Oct-TriA$_2$ (Aib3)

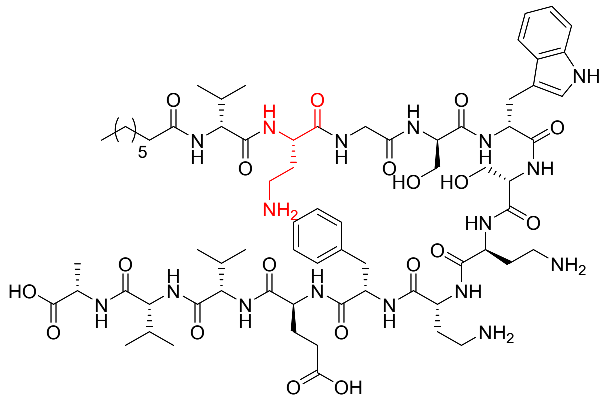

71. Oct-TriA$_2$ (Dab2)

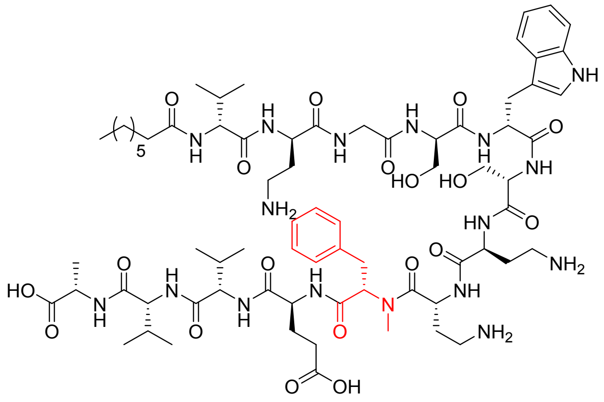

72. Oct-TriA$_2$ (*N*MePhe9)

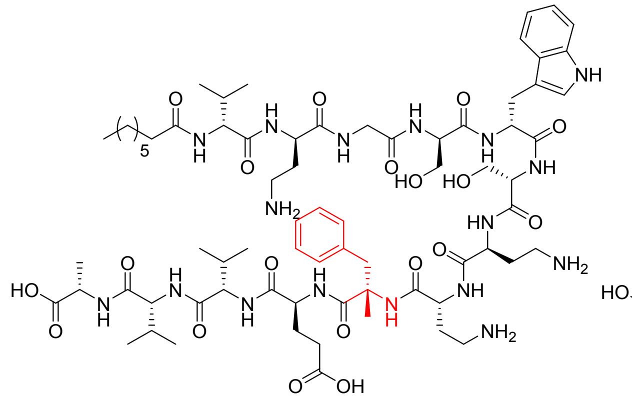

73. Oct-TriA$_2$ (αMePhe9)

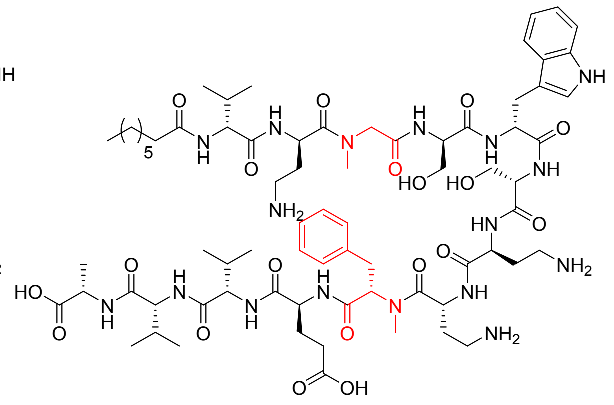

74. Oct-TriA$_2$ (Sar3, *N*MePhe9)

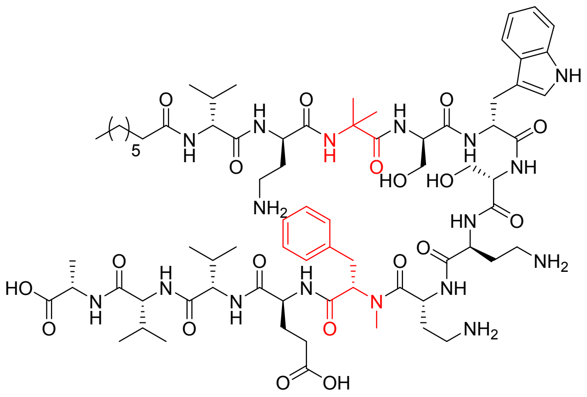

75. Oct-TriA$_2$ (Aib3, *N*MePhe9)

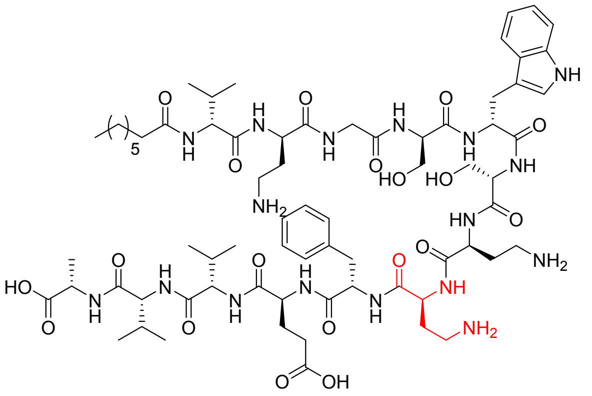

76. Oct-TriA$_2$ (Dab8)

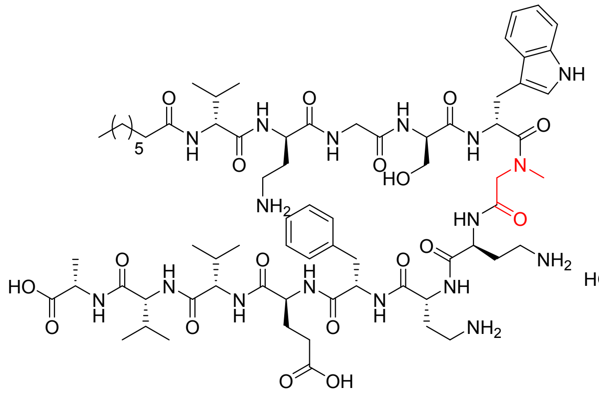

77. Oct-TriA$_2$ (Sar6)

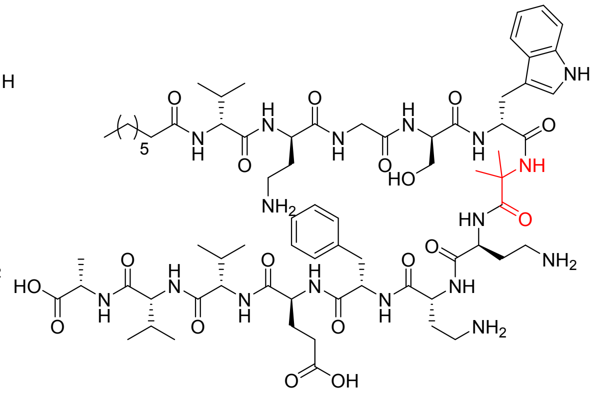

78. Oct-TriA$_2$ (Aib6)

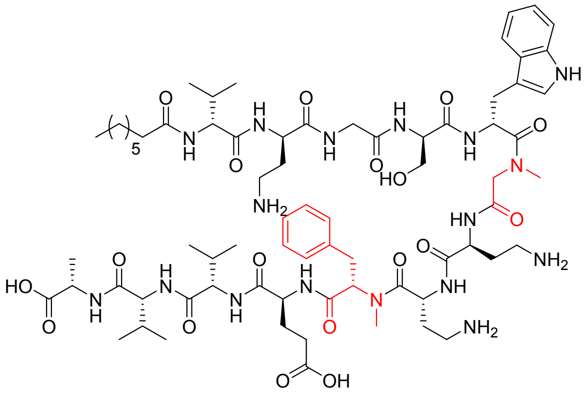

79. Oct-TriA$_2$ (Sar6, *N*MePhe9)

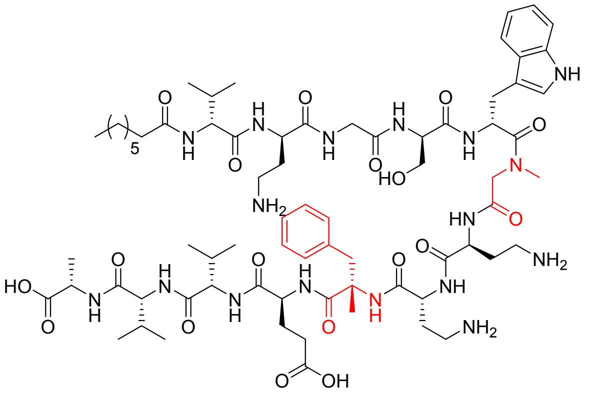

80. Oct-TriA$_2$ (Sar6, αMePhe9)

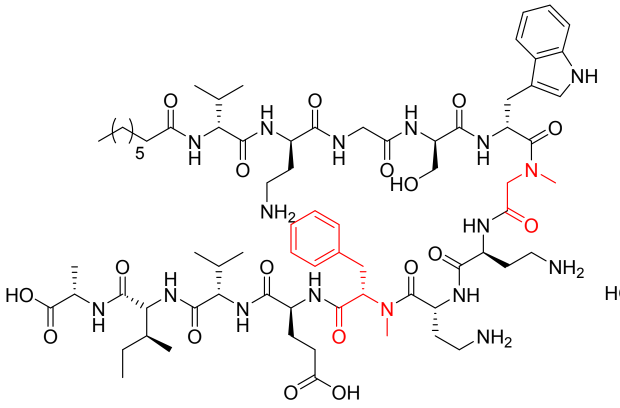

81. Oct-TriA$_1$ (Sar6, *N*MePhe9)

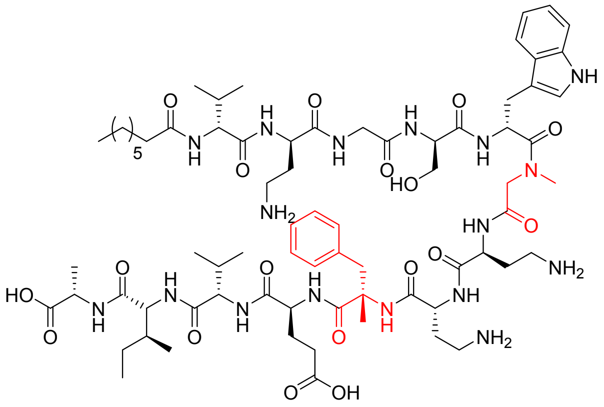

82. Oct-TriA$_1$ (Sar6, αMePhe9)

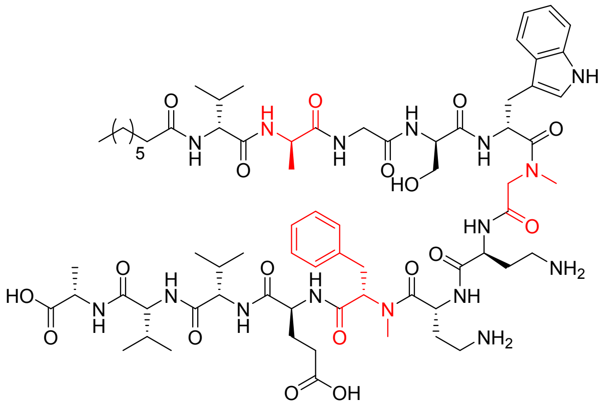

83. Oct-TriA$_2$ (D-Ala2, Sar6, *N*MePhe9)

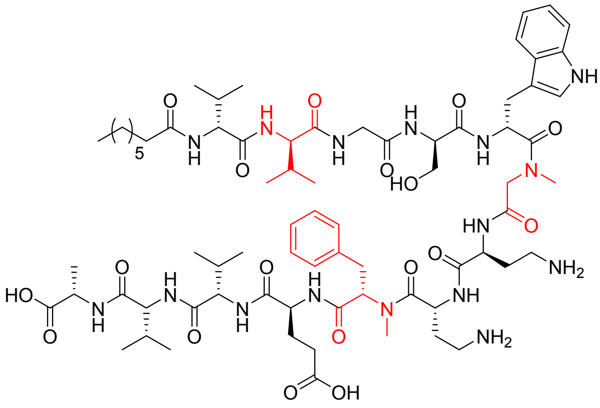

84. Oct-TriA$_2$ (D-Val2, Sar6, *N*MePhe9)

Table S9. MIC values of stabilised Oct-TriA$_1$ and A$_2$ analogues against representative Gram-negative and positive bacteria.

Bann, S. J.; Ballantine, R. D.; McCallion, C. E.; Qian, P.-Y.; Li, Y.-X.; Cochrane, S. A. J. Med. Chem. ***2019****, 62, 10466–10472.*

	MIC (μg/mL)	
**Analogue**	*E. coli* NCTC 12241	*S. aureus* NCTC 10788
**62**	1.56	50
**69**	>50	>50
**70**	>50	>50
**71**	>50	>50
**72**	6.25	>50
**73**	>50	>50
**74**	>50	>50
**75**	>50	>50
**76**	ND	ND
**77**	3.13	>50
**78**	3.13	>50
**79**	6.25	>50
**80**	>50	>50
**81**	3.13	>50
**82**	>50	>50
**83**	>50	>50
**84**	>50	>50

Table S10. Haemolytic activity of stabilised Oct-TriA$_2$ analogues.

Bann, S. J.; Ballantine, R. D.; McCallion, C. E.; Qian, P.-Y.; Li, Y.-X.; Cochrane, S. A. J. Med. Chem. ***2019****, 62, 10466–10472.*

**Analogue**[a]	**% haemolysis**[b]
**62**	53
**72**	30
**77**	38
**79**	60
**83**	78

[a] All peptides were tested at 100 µg/mL. [b] Haemolytic assays were run in triplicate. Absorbance of each sample was measured at 415 nm and percent haemolysis due to the corresponding peptide was calculated relative to Triton X-100 (taken as 100%).

A tridecaptin-based fluorescent probe for differential staining of Gram-negative bacteria
Wang, W.; Wang, Y.; Lin, L.; Song, Y.; Yang, C. J. *Anal. Bioanal. Chem.* **2019**, 411, 4017-4023.

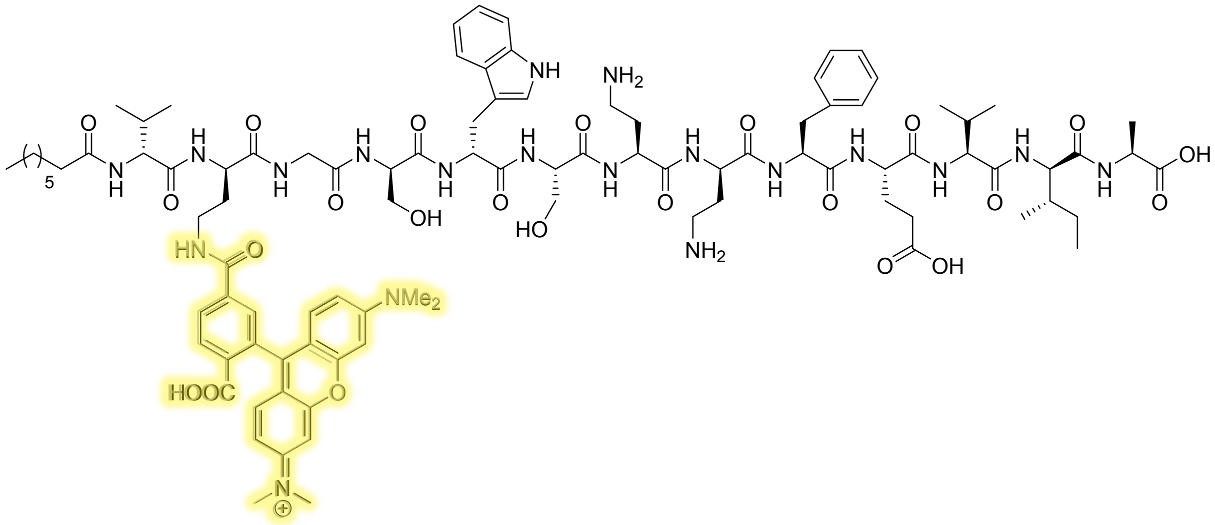

85. TriA-TAMRA

Studies on tridecaptin B_1, a lipopeptide with activity against multidrug resistant Gram-negative bacteria

Cochrane, S. A.; Lohans, C. T.; van Belkum, M. J.; Bels, M. A.; Vederas, J. C.

Org. Biomol. Chem. **2015**, 13 (21), 6073-6081.

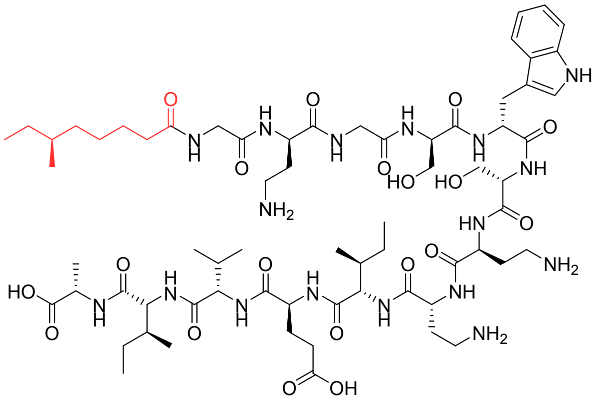

86. Natural $TriB_1$

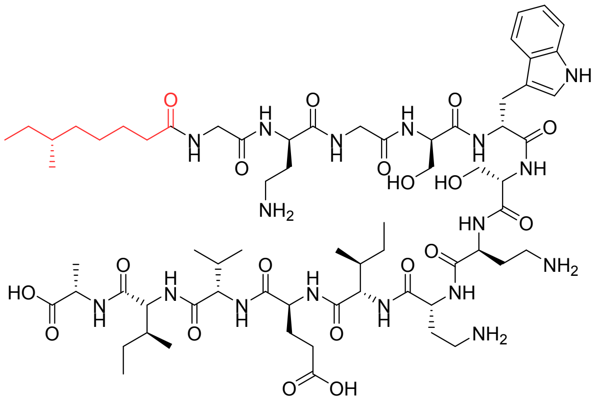

87. (6'*R*)-$TriB_1$

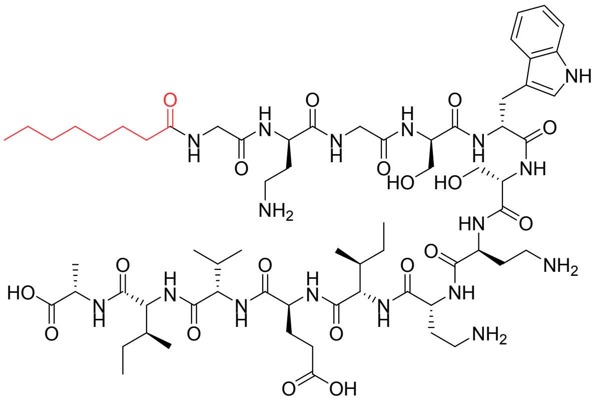

88. Oct-$TriB_1$

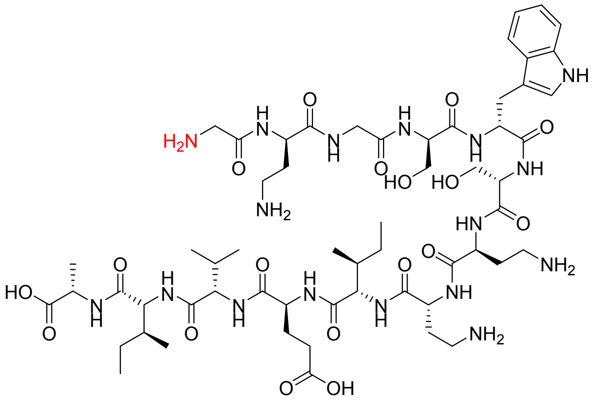

89. H-$TriB_1$

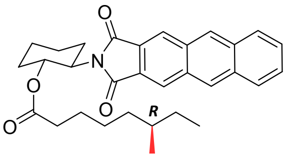

90. (6*R*)-methyl anthracenyl derivative

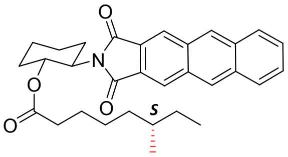

91. (6*S*)-methyl anthracenyl derivative

Table S11. MIC values of $TriB_1$ analogues against various bacterial strains.

Cochrane, S. A.; Lohans, C. T.; van Belkum, M. J.; Bels, M. A.; Vederas, J. C. Org. Biomol. Chem. ***2015****, 13 (21), 6073–6081.*											
	**MIC (μg/mL)**										
**Analogue**	*E. coli* ATCC 25922	*S. enterica* ATCC 13311	*P. aeruginosa* ATCC 27853	*K. pneumoniae* ATCC 13883	*K. pneumoniae* ATCC 700603	*A. baumannii* ATCC 19606	*A. baumannii* ATCC BAA 1605	*E. faecalis* ATCC 29212	*S. aureus* ATCC 29213	*Bacillus cereus* ATCC 21928	*Bacillus mycoides* ATCC 21929
**86**	6.25	3.13	12.5	6.25	3.13	25	25	>100	>100	>100	>100
**87**	12.5	6.25	25	6.25	6.25	50	25	>100	>100	>100	>100
**88**	12.5	12.5	25	12.5	6.25	50	50	>100	>100	>100	>100
**89**	>100	>100	>100	>100	>100	>100	>100	>100	>100	>100	>100

Tridecaptin M, a New Variant Discovered in Mud Bacterium, Shows Activity against Colistin- and Extremely Drug-Resistant *Enterobacteriaceae*
Jangra, M.; Kaur, M.; Tambat, R.; Rana, R.; Maurya, S. K.; Khatri, N.; Ghafur, A.; Nandanwar, H.
Antimicrob. Agents Chemother. **2019**, 63 (6), e00338-19.

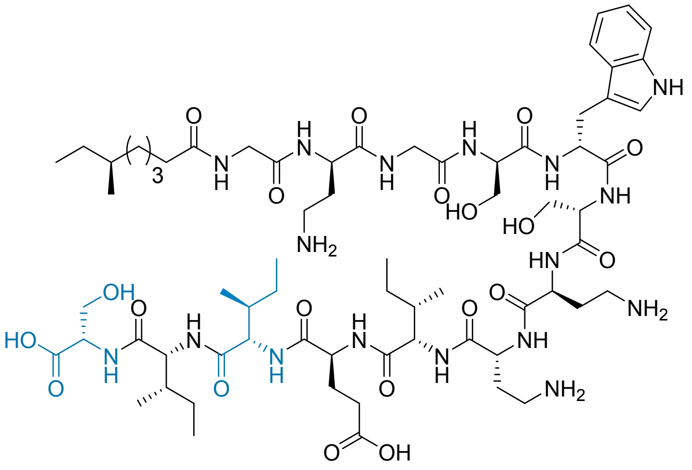

92. $TriM_1$

Table S12. MIC values of $TriM_1$ against various bacterial strains.

Jangra, M.; Kaur, M.; Tambat, R.; Rana, R.; Maurya, S. K.; Khatri, N.; Ghafur, A.; Nandanwar, H. Antimicrob. Agents Chemother. ***2019****, 63 (6), e00338-19.*	
**Strain**	**MIC (μg/mL)**
K. pneumoniae ATCC 700603	4
Kp ATCC BAA-1705	2
Kp ATCC BAA-1706	4
Kp ATCC BAA-2146	2
Kp ATCC 15380	1
Kp ATCC 29665	0.5
Kp subsp. *rhinoscleromatis* ATCC 13384	4
K. oxytoca MTCC 8295	2
Enterobacter aerogenes MTCC 10208	4
E. cloacae MTCC 509	4
E. coli ATCC 25922	4
Ec ATCC 35218	4
Ec 9062 (clinical isolate)	4
Ec 7932 (clinical isolate)	4
P. aeruginosa ATCC 27853	16
A. baumannii ATCC 19606	>32
S. enterica ATCC 10708	4
K. pneumoniae MDR (polymyxin-sensitive) (19 clinical strains ∴range)	2 - 8

Table S13. MIC values of $TriM_1$ against colistin-resistant *K. pneumoniae* and MDR *E. coli*.

Jangra, M.; Kaur, M.; Tambat, R.; Rana, R.; Maurya, S. K.; Khatri, N.; Ghafur, A.; Nandanwar, H. Antimicrob. Agents Chemother. ***2019****, 63 (6), e00338-19.*	
**Strain**	**MIC (μg/mL)**
K. pneumoniae (clinical isolates)	
AH-1	2
AH-2	2
AH-3	2
AH-4	2
AH-5	2
AH-6	2
AH-7	2
AH-8	2
AH-9	2
AH-10	2
AH-11	2
AH-12	2
AH-13	2
AH-14	2
AH-15	2
AH-16	2
AH-17	2
AH-18	2
AH-19	2
E. coli (food isolates)	
CF-23	4
CF-45	4
CF-47	4

Table S14. Toxicity data for $TriM_1$.

Jangra, M.; Kaur, M.; Tambat, R.; Rana, R.; Maurya, S. K.; Khatri, N.; Ghafur, A.; Nandanwar, H. Antimicrob. Agents Chemother. ***2019****, 63 (6), e00338-19.*		
**Analogue**	**% haemolysis**[a]	**IC_{50} (μg/mL)**[b]
**92**	0	>250

[a] Haemolysis at 100 μg/mL. However, >50% haemolysis was observed at a peptide concentration of 200 μg/mL. Assays were run in triplicate, with two biological repeats, and absorbance of each sample was measured at 570 nm while percent haemolysis due to the corresponding peptide was calculated relative to Triton X-100 (taken as 100%). [b] Peptide concentrations of 0 – 250 μg/mL were tested against HEK 293 and J774 cell lines. Assays were run in triplicate and compared to PBS standard (taken as 100%).

Purification and biological activity of natural variants synthesized by tridecaptin M gene cluster and in vitro drug-kinetics of this antibiotic class

Jangra, M.; Kaur, M.; Podia, M.; Tambat, R.; Singh, V.; Chandal, N.; Mahey, N.; Maurya, N.; Nandanwar, H. *Sci. Rep.* 2019, 9 (18870), doi.org/10.1038/s41598-019-54716-8.

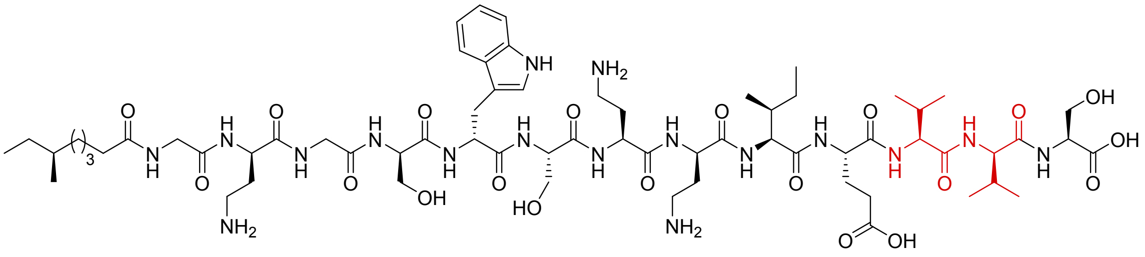

93. $TriM_2$

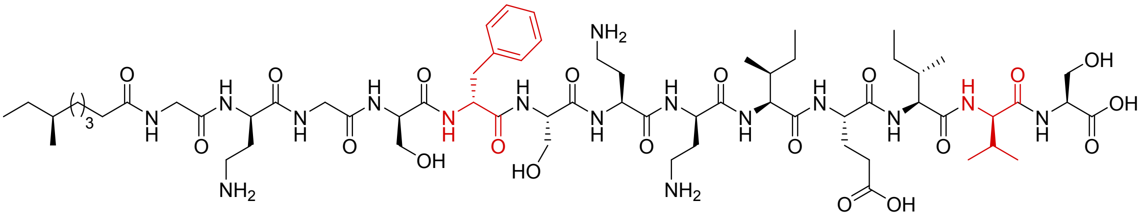

94. $TriM_5$

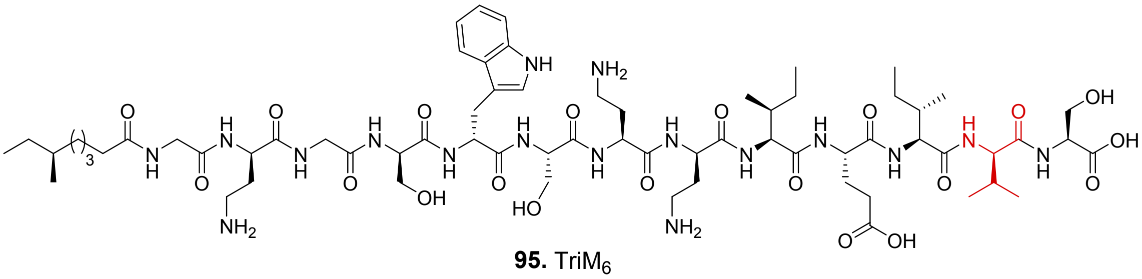

95. $TriM_6$

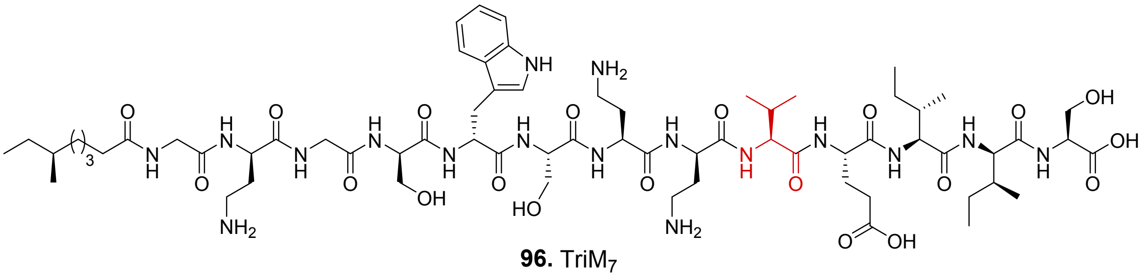

96. $TriM_7$

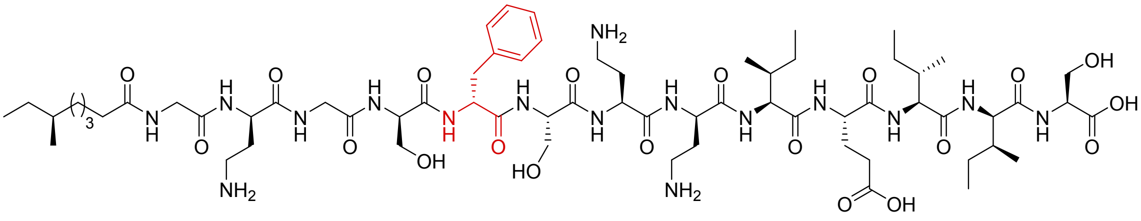

97. $TriM_8$

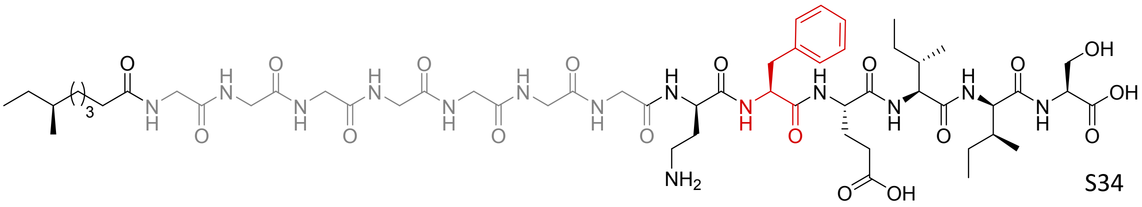

98. $TriM_{11}$:

Residues 1-7 could not be conclusively determined

Table S15. MIC values of $TriM_1 – M_{11}$ analogues against various bacterial strains.

Jangra, M.; Kaur, M.; Podia, M.; Tambat, R.; Singh, V.; Chandal, N.; Mahey, N.; Maurya, N.; Nandanwar, H. Sci. Rep. 2019, 9 (18870), *doi.org/10.1038/s41598-019-54716-8.*

**Analogue**	**MIC (μg/mL)**										
	K. pneumoniae ATCC 700603	*K. pneumoniae AH-3 (Col-R)*	*K. pneumoniae AH-16 (Col-R)*	*E. coli* CF-23 (*mcr-1*)	*P. mirabilis* MTCC 1429	*Serratia marcescens MTCC 97*	*K. pneumoniae* P3R (M1-R)	*K. pneumoniae* GMCH 13	*K. pneumoniae* GMCH 15	*A. baumannii* ATCC 19606	*P. aeruginosa* ATCC 27853
**92**	4	4	4	4	>16	8	64	8	16	64	>128
**93**	8	8	>16	16	>16	>16	>32	ND	ND	>32	>32
**94**	16	16	16	16	16	8	16	ND	ND	>32	>32
**95**	4	4	2	4	>16	16	16	ND	ND	>32	>32
**96**	8	8	8	16	>16	8	16	ND	ND	>32	>32
**97**	4	8	4	4	>16	16	128	ND	ND	128	>128
**98**	2	1	1	1	4	4	8	4	2	4	8

Table S16. Haemolytic activity of $TriM_1$ – $TriM_{11}$ analogues.

Jangra, M.; Kaur, M.; Podia, M.; Tambat, R.; Singh, V.; Chandal, N.; Mahey, N.; Maurya, N.; Nandanwar, H. Sci. Rep. 2019, 9 (18870), doi.org/10.1038/s41598-019-54716-8.	
**Analogue**[a]	**% haemolysis**[b]
**92**	10
**93**	2
**94**	0
**95**	17
**96**	1
**97**	78
**98**	50

[a] Peptide concentration of 128 µg/mL. [b] Percent haemolysis due to the corresponding peptide was calculated relative to Triton X-100 (taken as 100%). Exact data points were not provided therefore values were approximated from the graph provided.
